# Study of multi-size proppant breakage mechanism under deep reservoir conditions for enhanced geothermal system development

**DOI:** 10.1038/s41598-025-21180-6

**Published:** 2025-10-24

**Authors:** Buge Du, Guangqing Zhang, Ruiheng Jin, Junfeng Zhu, Dawei Zhou

**Affiliations:** 1 Department of Petroleum Engineering, China University of Petroleum-Beijing, Beijing, 102249 China; 2 National Key Laboratory of Petroleum Resources and Engineering, China University of Petroleum-Beijing, Beijing, 102249 China

**Keywords:** Proppant breakage, Proppant deformation, Multi-size proppants, Deep reservoir, Visualization, Geothermal energy, Petrol, Ceramics

## Abstract

Proppant breakage is a significant concern in fracturing treatments in deep reservoirs, reducing the efficiency of enhanced geothermal systems (EGS). However, little research has been conducted on the breakage process of proppants under high closure stress conditions. A visualization experiment was designed to investigate processes of proppant breakage and layer deformation in real-time under 100 °C and high closure stress (0–50 MPa) conditions. We observed significant non-uniform breakage in the multi-size proppant layer, which resulted in large-size pores with high connectivity in the regions of low breakage degree, potentially maintaining permeability. Moreover, the non-uniform breakage forms a composite structure in two stages. This process mitigates subsequent proppant breakage, thereby preserving the non-uniform breakage pattern and maintaining a locally high-connectivity porous structure. In addition, we discussed the nonlinear deformation and stiffness of the proppant layer under stress loading. Finally, the applicability of the conclusions has been demonstrated by comparing granite-proppant experiments with the same conditions. The non-uniform breakage characterization of multi-size proppant layer in the granite-proppant experiments was observed as well. This research reveals the breakage mechanism of the multi-size proppant, which is expected to facilitate the optimization of proppant placements in deep fracturing operations.

## Introduction

With the development of sustainable energy sources exploitation technology and the growth of environmental awareness, the focus of researchers and engineers has shifted from conventional reservoirs to the abundant resources of deep reservoirs (≥ 4500 m). In particular, natural geothermal reservoirs of hard and tight rocks, such as granite, have been exploited to harvest renewable and sustainable energy^1^. Geothermal energy has gained significant attention as a renewable source due to its high conversion efficiency and operational stability. Current extraction primarily relies on hydraulic circulation systems, where fluid is injected into subsurface reservoirs to enable continuous heat recovery through production wells^2,3^. However, geothermal energy extraction faces major geological challenges, particularly the low permeability of deep rock formations. Enhanced geothermal systems (EGS) address this by artificially stimulating reservoirs to increase permeability and improve energy recovery efficiency^3,4^.

Hydraulic fracturing is one of the most important stimulations for enhancing the efficiency of geothermal energy conversion by inducing artificial fractures and improving the permeability of medium fluids like water in rock masses. The sustainable maintenance of hydraulically induced fracture networks in EGS necessitates strategic proppant injection to counteract fracture closure caused by substantial in-situ stresses^5–9^.

**Fig. 1:**
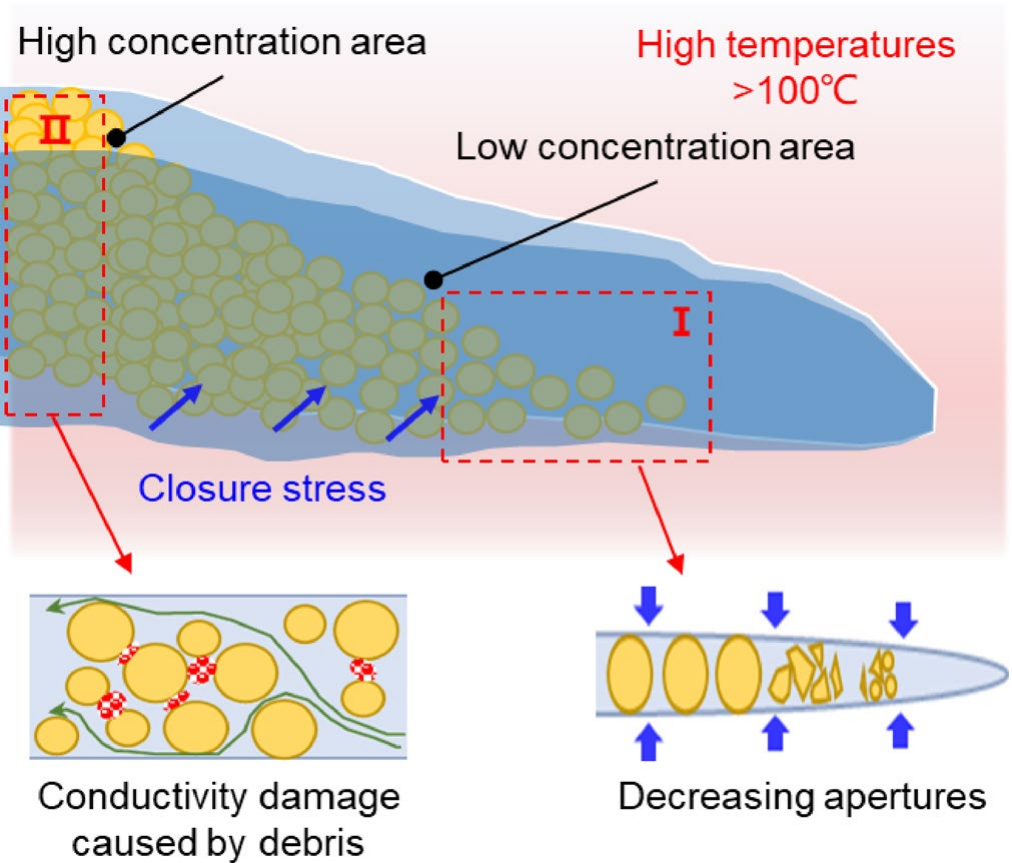
Effects of proppant breakage in hydraulic fractures within deep reservoirs.

The development of EGS is fraught with numerous difficulties, largely attributable to the presence of complex conditions such as high temperatures and stresses. Therefore, some scenarios seriously limit the efficacy of hydraulic fracturing, including fracturing fluid damage, low sand concentrations, and proppant breakage^10,11^. Moreover, with the propagation of hydraulic fracture, apertures gradually reduce, resulting in lower concentrations of proppants in regions that are farther from the perforation^12^. In these regions, proppant breakage occurs significantly (Region I in Fig. 1), with large amounts of proppant debris migrating and obstructing flow pathways (Region II in Fig. 1), leading to decreased fracture conductivity. Furthermore, the significant thermochemical environment in reservoirs can accelerate the proppant diagenesis process^13,14^.

Despite the proposal of certain fracturing technologies devoid of proppants by some researchers, which rely on self-support to ensure a certain conductivity and to reduce costs, the utilization of proppants remains essential for deep reservoirs, where high stresses induce severe fracture closure^15,16^. Bijay et al. confirmed that the use of sintered bauxite particles as proppants is expected to enhance the fracture conductivity. However, due to proppant breakage and other behaviors, the effectiveness of EGS will significantly decrease over time^7^. Current research primarily identifies the mechanisms and factors that affect proppant breakage under geothermal reservoir conditions, to optimize the proppant performance^17–19^. Specifically, the research of proppant breakage should incorporate its structure characterizations and rock contacts into the analysis, based on the material yield criterion^20,21^. For instance, the efficacy of improving the material strength, reducing the size of proppants, and coating has been demonstrated^22–26^. In addition, researchers are no longer limited to the proppant pack in the macroscopic scale, and microscopic breakage processes can be captured to study the damage mechanism^27–30^. Nevertheless, it is difficult to obtain real-time information on the deformation and breakage processes of proppants under high stresses. A significant number of current studies have been grounded in obtaining qualitative insights through optical observations after testing^31–34^. As a result, the conclusions drawn from this method may necessitate further validation.

**Fig. 2:**
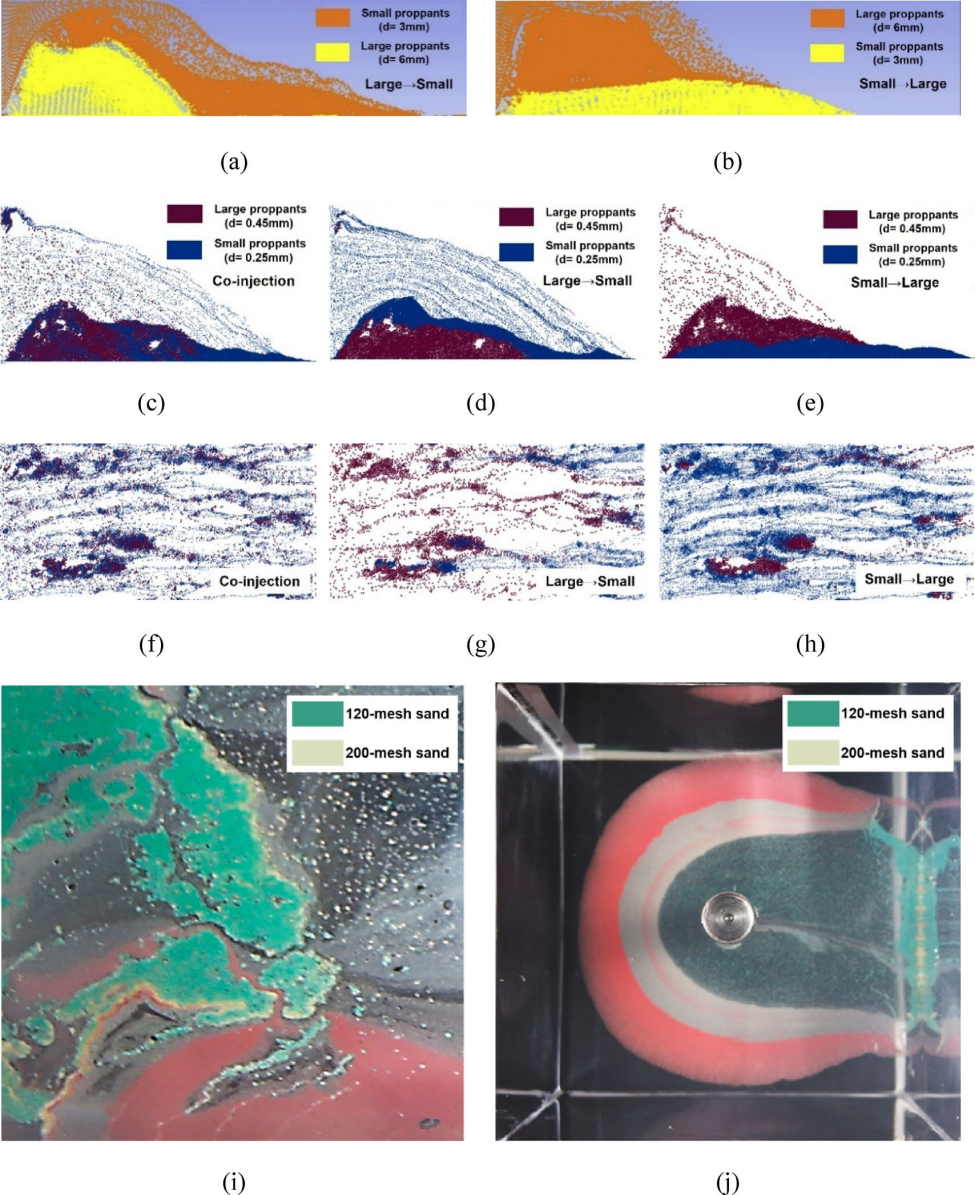
The distribution of multi-size proppants with different pumping sequences, fracture roughness, and fracture types. The results are obtained from the studies^36–38^.

Furthermore, to increase the proppant placement area in branch fractures with various apertures, some researchers have proposed and optimized the pumping schedule of multi-size proppant^35–38^. Through numerical and experimental methods, they investigated the transport and final distribution of multi-size proppants under different pumping sequences, fracture geometry, and fracture types. The results demonstrate that when the fractures are straight, the mixing feature of the different proppants is insignificant, with the distribution areas non-overlapping (Fig. 2a,b, and j). However, the actual fracture geometry is more complex, and the effects of the tortuosity on the proppant transport cannot be disregarded. It is manifested as the mixing of large and small proppants occurring in the vertical tortuous fracture under certain pumping sequences (Fig. 2c,i). In contrast, proppants in the horizontal tortuous fracture are distributed in clusters while the mixing phenomenon presents more obviously (Fig. 2f–h). Consequently, the multi-size proppant distribution in the actual hydraulic fracture is non-uniform, with the mixing phenomenon of different-size proppants being prevalent, under which the proppant breakage and fracture closure behaviors will be more complicated as well.

We firmly believe that current research must address three critical issues: (1) the characteristics of proppant breakage under low concentrations in deep-hard rock reservoirs; (2) the evolution of the proppant layer’s pore structure with increasing breakage; and (3) the effects of size ratios in multi-size proppant placement on breakage, layer deformation, and pore structure evolution.

In this paper, we designed a visualization experiment to study processes of proppant breakage in real-time under deep and hard rock reservoir conditions (0–50 MPa, 100 °C). Four major research contents were carried out as follows:

- Based on the visual method, the breakage process of multi-size proppants was captured.
- The correlation between the deformation of the proppant layer and the breakage mechanism of multi-size proppant was established.
- The characterization of interparticle pores and the evolution of pore volumes in the proppant layer was clarified.
- Advanced discussions about the limitations of the visual method were stated, and the reliability of our results was validated in the particular condition.

## Methods

### The introduction to the visual experiment

**Fig. 3:**
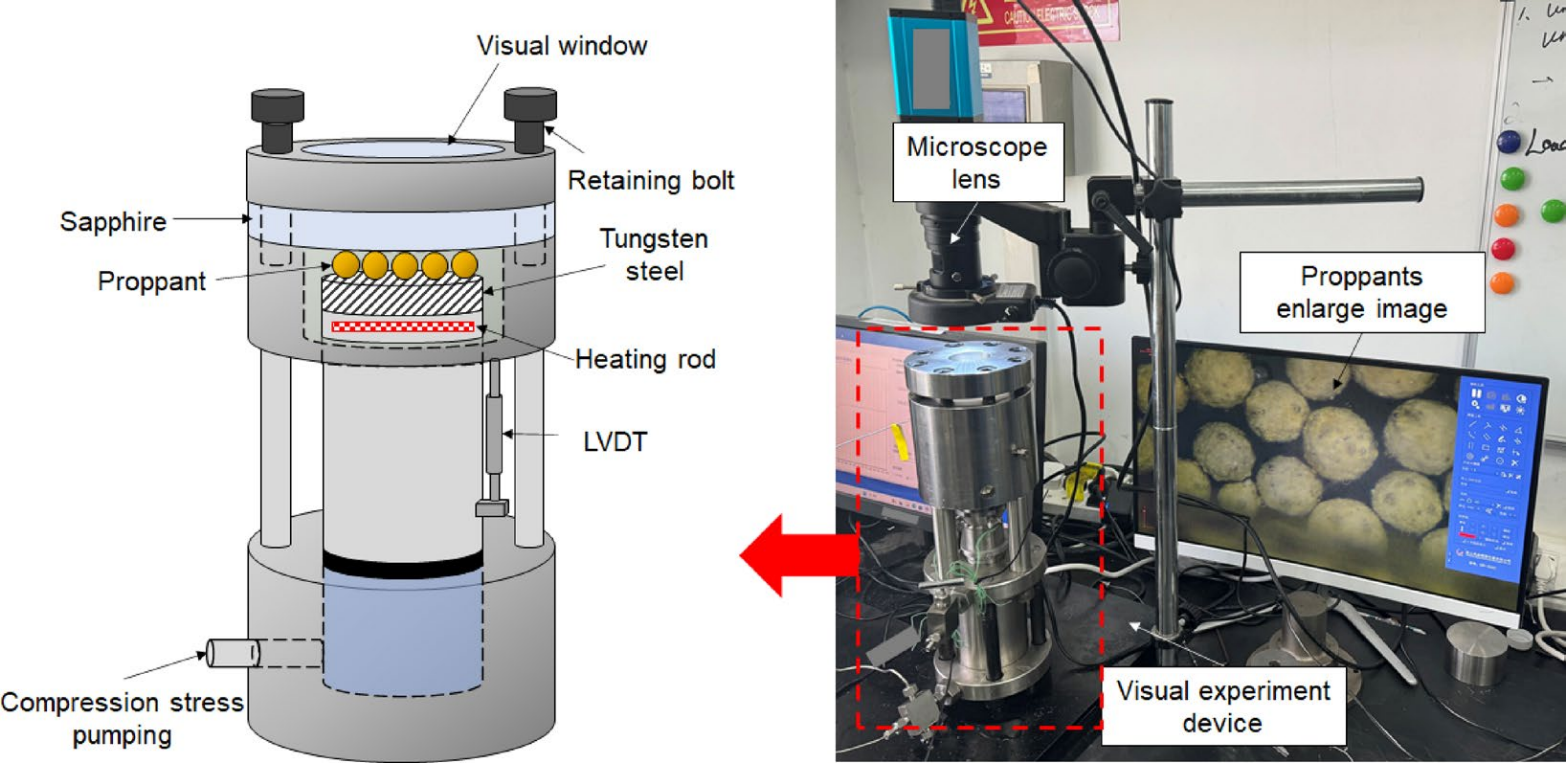
The visual experimental system of proppant breakage.

We designed a visual experimental method to capture the proppant breakage image in real time (Fig. 3). The visualized experiment device primarily consists of a hydraulically driven piston (capable of providing 0–50 MPa), a high-transparency sapphire window (light transmittance > 90%), a microscope (magnification range of 1–7×), and an LVDT (measurement range of 0.01–4 mm with an accuracy of 0.01 mm). Using the axial movement of the piston pump, the specimen was exerted to compressive stress. To obtain clear optical images of the proppants, we selected a transparent and high-strength sapphire above the specimen. An optical microscope was placed above the visualization window to record the proppant images at loading times. The field of view of the microscope covers a square area of 11 mm × 11 mm at the center of the specimen.

The mixing ratio of multi-size proppants varies across different conditions, it is anticipated that the hydraulic fracture complexity increases induced by natural fracture interactions in the reservoir, leading to a reduction in the proportion of larger-size proppants within the fracture tip^37^. Therefore, to investigate the fracture closure and pore damage behavior of the proppant pack caused by proppant breakage in various conditions is necessary. To simulate the mixing characterization of multi-size proppants in the hydraulic fracture, five proppant placement patterns with different ratios of 20/40 mesh and 40/70 mesh low-density ceramic proppant (LDC) were conducted. The elastic modulus of the ceramic proppant is about 37.2 GPa, its hardness is about 15.9 GPa.

**Fig. 4:**
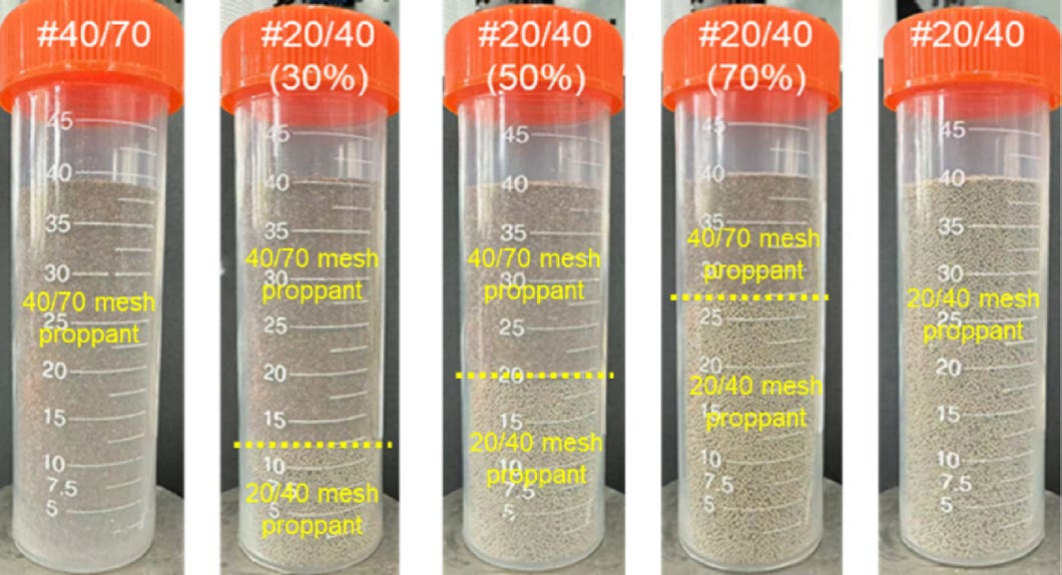
Varying ratios of 20/40 mesh and 40/70 mesh proppants (Before mixing).

Using the ratio of 20/40 mesh proppant as the single variable, the ratios were set at 0% (pure 40/70 mesh proppant), 30%, 50%, 70%, and 100% (pure 20/40 mesh proppant) (Fig. 4). This ratio gradient design, transitioning from pure small-sized to pure large-sized proppants (with interval gradients of 30%, 20%, 20%, and 30%), systematically characterizes the mechanism of multi-size proppant ratios on proppant breakage. Following a thorough mixing process involving ceramics of different sizes, a uniform monolayer proppant was placed on the surface of a steel cylindrical plug (diameter: 25 mm, height: 12.5 mm).

While this study simplifies the proppant placement to facilitate mechanistic analysis and high-resolution visualization, it does not fully replicate the multi-layer proppant distribution in near-wellbore regions of field-scale hydraulic fractures. Investigation of multi-layer proppant breakage will be addressed in future research. The experiment has the advantage of capturing images of proppant breakage in real-time and analyzing the evolution of pore structure in the proppant layer. However, two key controversies surrounding the visualization device coexist: Is the contact between sapphire-proppant-steel similar to that of proppant-rock? Why was the steel used instead of the real rock? Both issues are discussed in “Discussions” section to ensure the analogy and reliability of our experimental results.

Table 1 illustrates the experimental parameters. The specimens correspond to five placement patterns, categorized into two types: the single-size range of proppants-placed specimens (SP) and the multi-size proppants-placed specimens (MP). The proppant concentration slightly increased with the ratio of 20/40 mesh proppants enhancing. Additionally, the experimental temperature was set at 100 °C.

**Table 1:** The experimental parameter.

Specimen ID	Specimen type	Volume ratio	Concentration (kg/m^2^)	Temperature (°C)
#40/70	SP	40/70: 100%, 20/40: 0%	0.42	100
#20/40(30%)	MP	40/70: 70%, 20/40: 30%	0.44	
#20/40(50%)		40/70: 50%, 20/40: 50%	0.45	
#20/40(70%)		40/70: 30%, 20/40: 70%	0.48	
#20/40	SP	40/70: 0%, 20/40: 100%	0.5	

### The distributions of proppant diameter

**Fig. 5:**
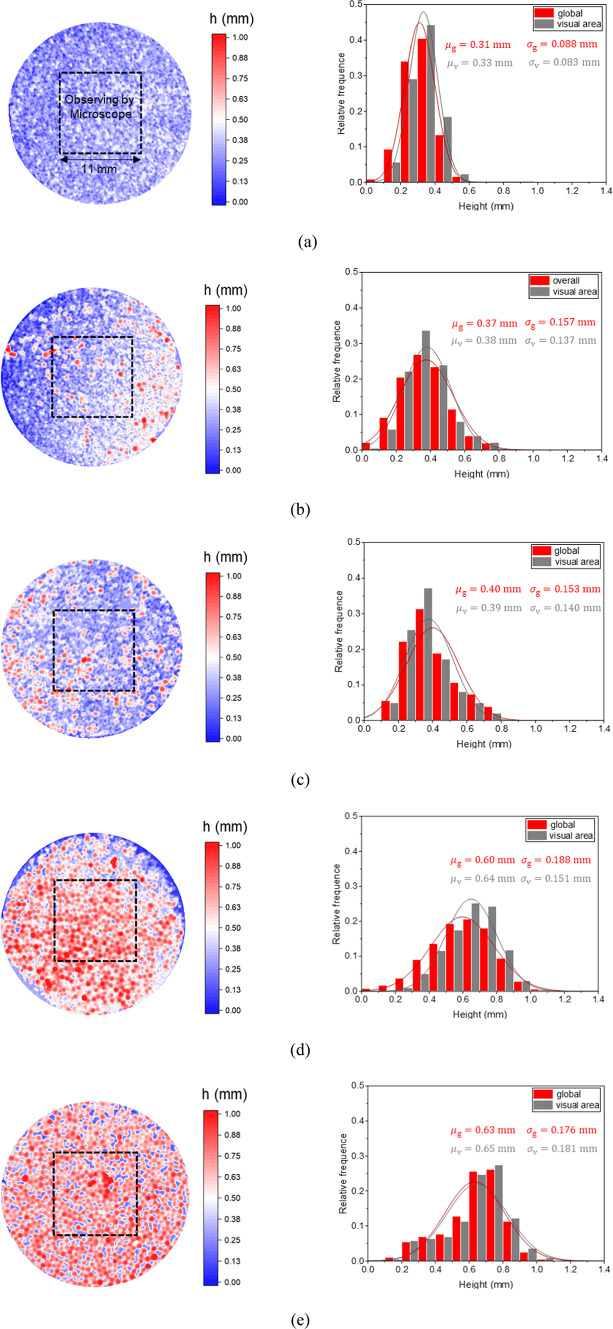
The 3D scanning results of five proppant-placed specimens (left). Comparative analysis of global height distribution and microscopic visual areas (right). $\mu_{{\text{g}}}$ is the mean of the global height of the specimen, $\mu_{{\text{v}}}$ is the mean of the height of the observation area, $\sigma_{{\text{g}}}$ is the standard deviation of the global height of the specimen, $\sigma_{{\text{v}}}$ is the standard deviation of the height of the observation area. (**a**) #40/70; (**b**) #20/40(30%); (**c**) #20/40(50%); (**d**) #20/40(70%); (**e**) #20/40.

To evaluate the statistical representativeness of microscopically observed areas in proppant-placed specimens, this study systematically acquired full-field surface topography data using three-dimensional laser scanning technology (resolution ≈ 0.01 mm). A comparative spatial distribution analysis was conducted between the global specimen data and microscopic observation areas (Fig. 5). Parametric characterization through normal distribution modeling revealed that the mean difference range was 2.5–6.7%, and the standard deviation fluctuation amplitude was 2.5–19.7%. These results systematically demonstrate that the local observation areas under microscopy effectively represent the global statistical features of proppant placement, and confirm the validity of partial investigations for characterizing global proppant breakage behavior.

### The loading setup

Setup 1: Before conducting the formal experiment, adjust the position of the plunger to make the proppant and sapphire come into contact. Record the value of the LVDT at this moment as the initial aperture. The initial aperture approximates the maximum proppant diameter, which can be independently quantified via 3D scanning.

**Fig. 6:**
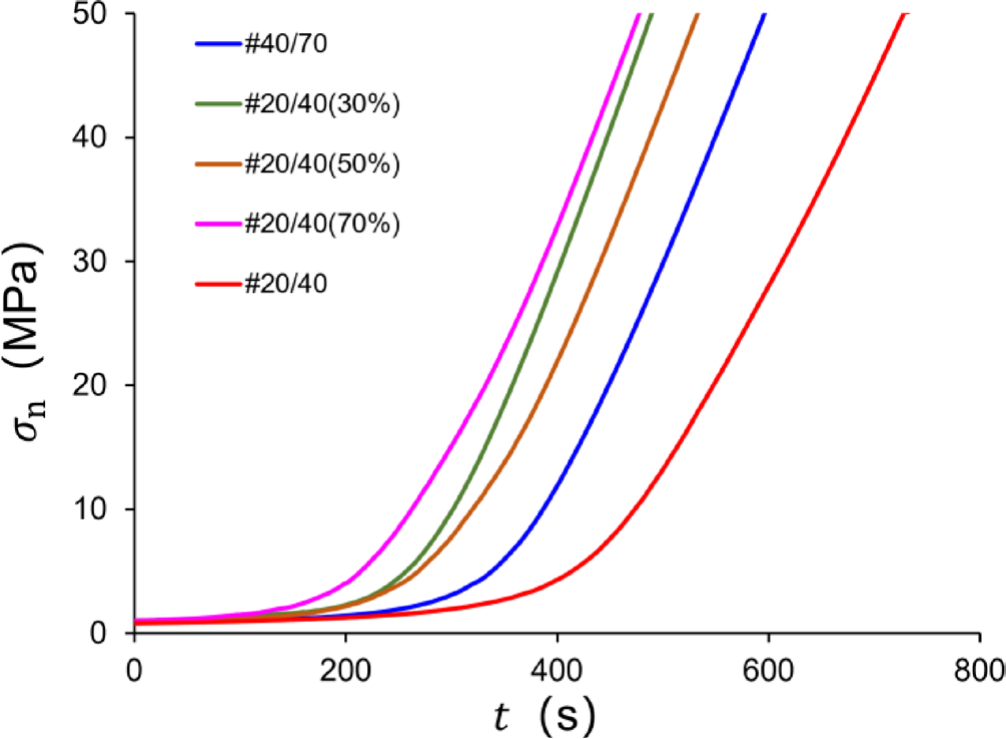
The loading curves of experiments.

Setup 2: Kerosene was injected into the plunger pump at a constant rate (1 mL/min) to exert closure stresses on the rock specimen. Simultaneously, the deformation data of the proppant layer from the LVDT and microscope was recorded simultaneously until the closure stress reached 50 MPa. The curves of closure stress corresponding to the loading time can be seen in Fig. 6.

Setup 3: Following experimental execution, systematic analysis of microscopic imaging datasets and LVDT recordings was performed. The methodological framework governing image quantification and displacement data processing will be comprehensively detailed in later sections.

### The image processing

**Fig. 7:**
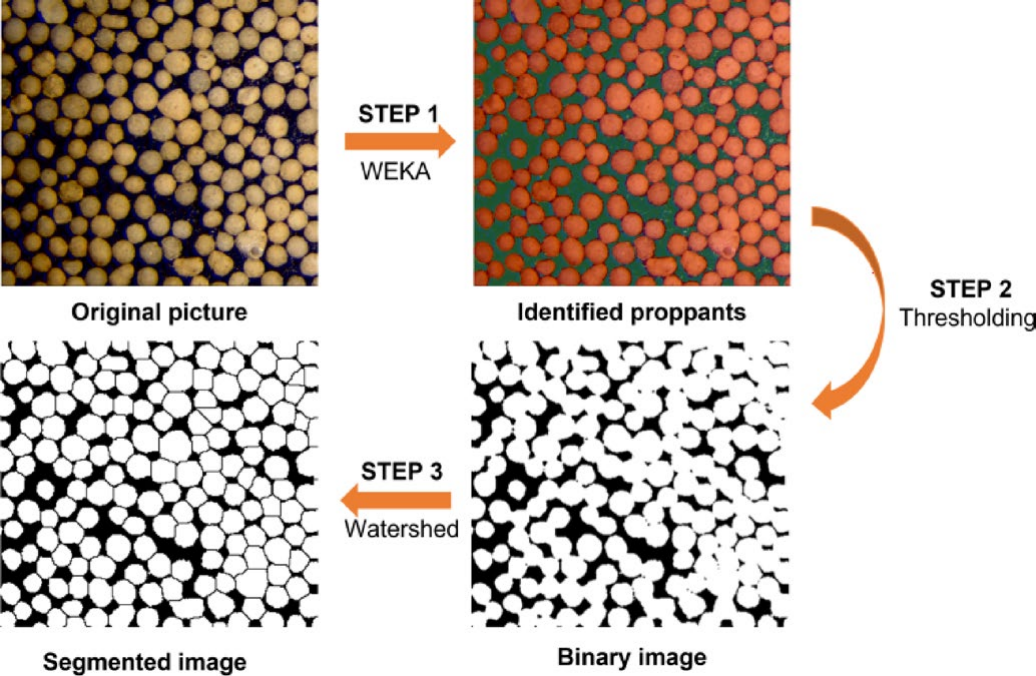
The processing flow of proppant breakage images.

Microscopic images were processed as Fig. 7. To obtain such pore size or projection area, the Weka Segmentation plugin of the ImageJ software was utilized to identify proppants in the original image (identified as the red area). The proppant particles were then extracted from the binary images. Finally, the particles were segmented by implementing a watershed operation.

## Results

### The process of proppant breakage

**Fig. 8:**
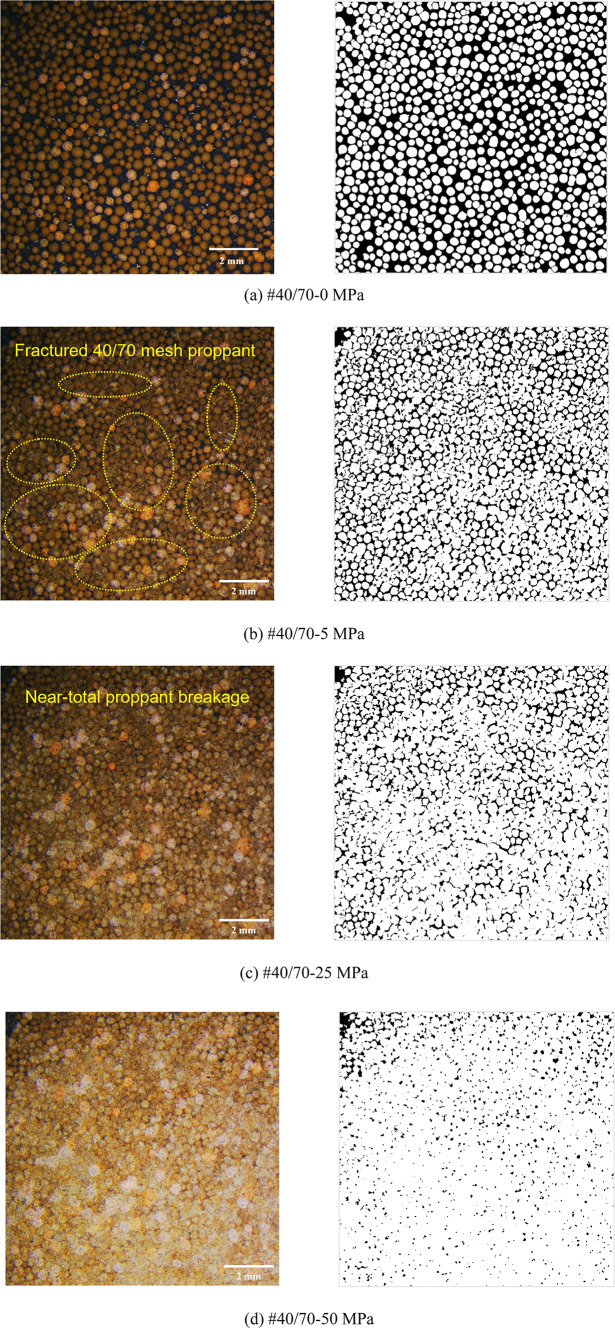
Proppant breakage physical images (left) and binary images (right) of #40/70.

**Fig. 9:**
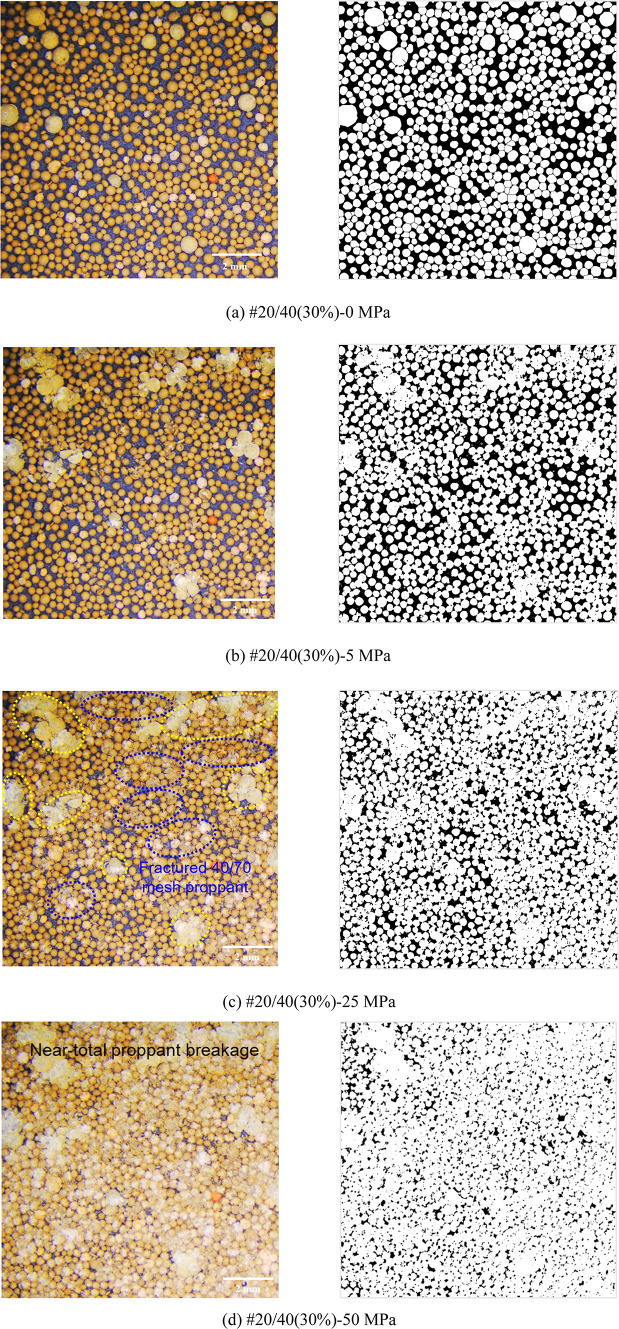
Proppant breakage physical images (left) and binary images (right) of #20/40(30%).

**Fig. 10:**
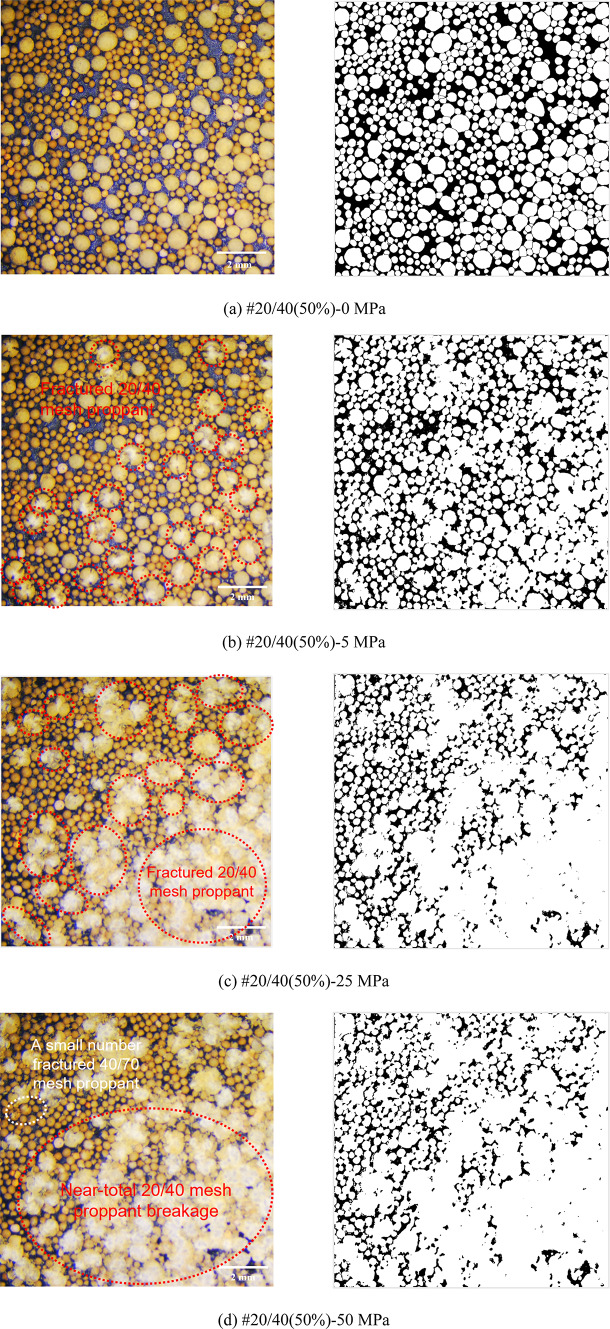
Proppant breakage physical images (left) and binary images (right) of #20/40(50%).

**Fig. 11:**
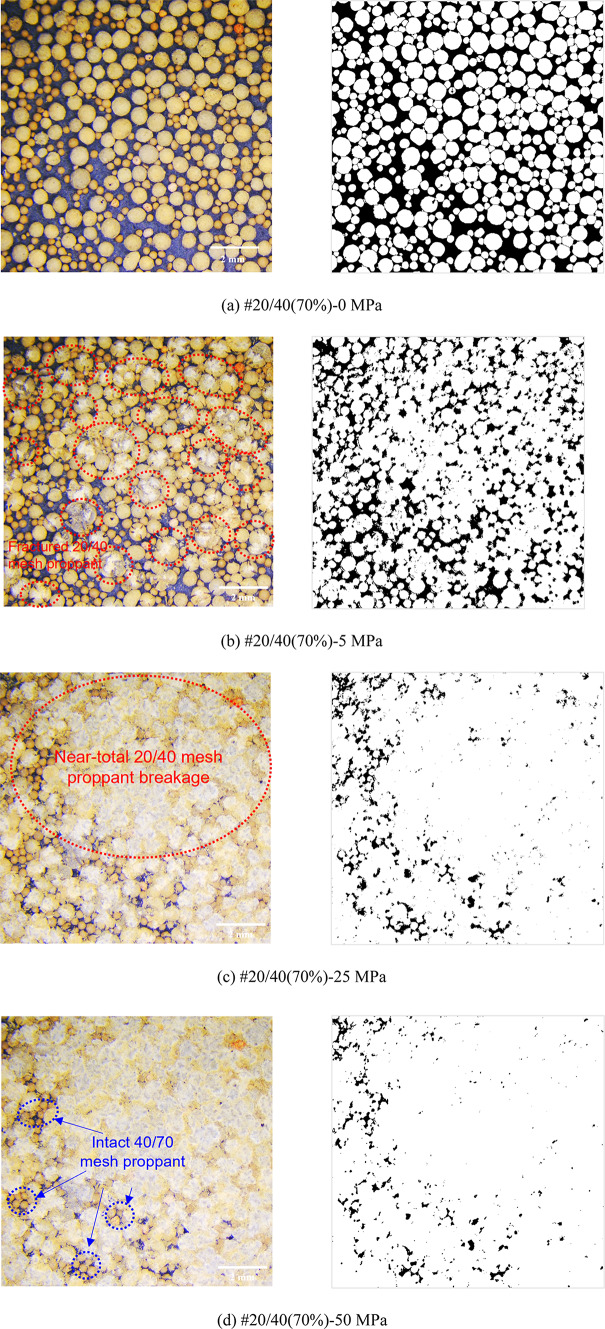
Proppant breakage physical images (left) and binary images (right) of #20/40(70%).

**Fig. 12:**
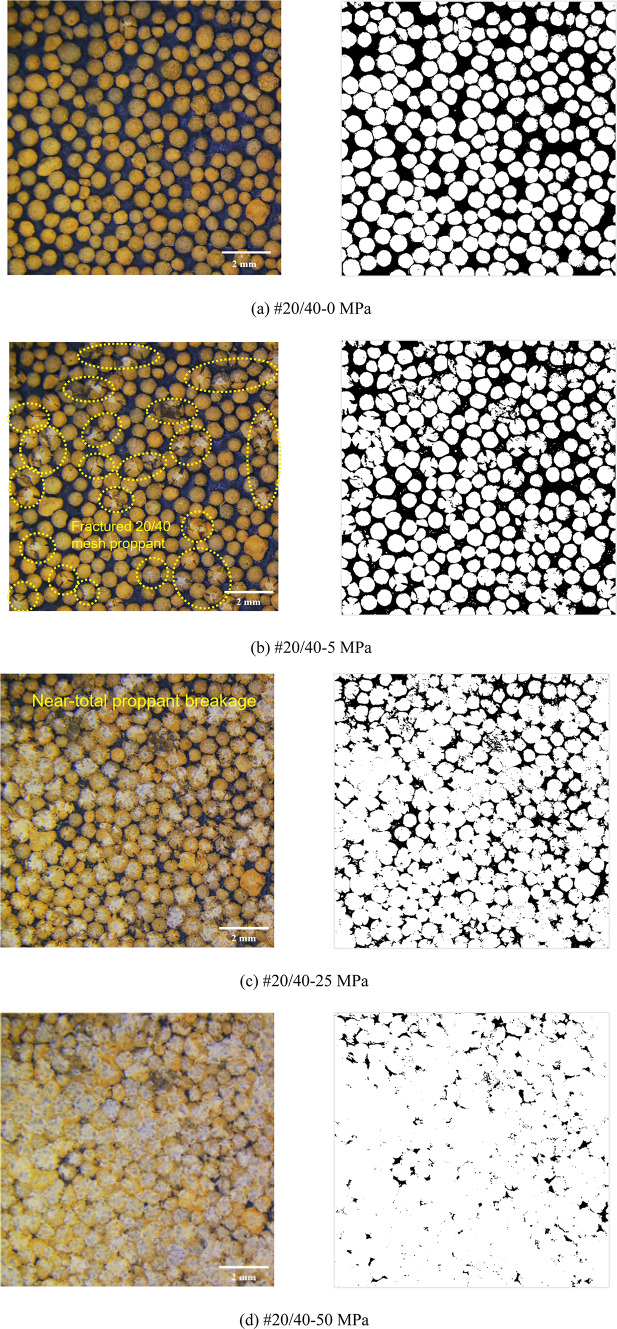
Proppant breakage physical images (left) and binary images (right) of #20/40.

Figures 8, 9, 10, 11 and 12 demonstrate images of proppant breakage at closure stresses of 0 MPa, 5 MPa, 25 MPa, and 50 MPa respectively. For the SP (#40/70 and #20/40), only a small portion of proppants break at 5 MPa (Figs. 8a and 12a), while almost all proppants are crushed at 50 MPa (Figs. 8d and 12d). However, a different phenomenon is observed in the MP: a significant non-uniform breakage occurs with the closure stress increasing. Under low closure stress conditions (less than 5 MPa), 20/40 mesh proppants are broken, followed by the failure of 40/70-mesh proppants (Figs. 9, 10, 11 and 12). Notably, as the closure stress progressively increases to 50 MPa, the number of broken 40/70 mesh proppants in #20/40(50%) become significantly lower compared to #40/70 (Figs. 8d and 10d). Additionally, fragments of crushed 20/40 mesh proppants are compressed, inducing radial deformations and contacting or even covering the adjacent smaller proppants (Fig. 10d).

Moreover, the proppant axial projection area increases significantly with proppant breakage. We can quantitatively discuss the proppant breakage severity level through image analysis. Thus, the proppant breakage degree $\Omega$ is defined as follows:

1 $$\begin{array}{*{20}c} {\Omega = \frac{{A_{{\text{c}}} - A_{{{\text{c}}0}} }}{{A_{{{\text{c}}0}} }} } \\ \end{array}$$

where $A_{{\text{c}}}$ is the proppant projection area at the current closure stress, mm^2^; $A_{{{\text{c}}0}}$ is the proppant projection area at 0 MPa, mm^2^.

**Fig. 13:**
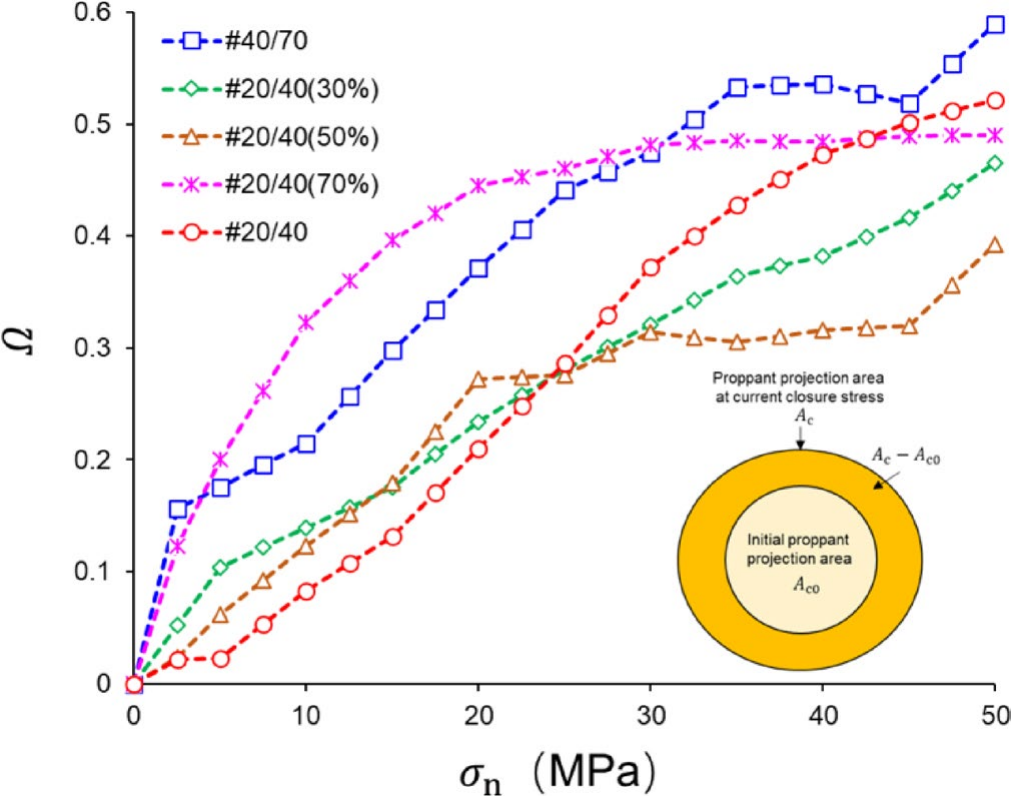
$\Omega$ of different specimens corresponding to $\sigma_{{\text{n}}}$.

Figure 13 illustrates $\Omega$ values of different patterns corresponding to the closure stress $\sigma_{{\text{n}}}$. It is evident that as $\sigma_{{\text{n}}}$ increases, there is a corresponding increase in proppant breakage, resulting in a larger $\Omega$. Moreover, when $\sigma_{{\text{n}}}$ reaches 50 MPa, the value of $\Omega$ of the SP outweighs that of the MP. In addition, the $\Omega$ of #40/70 is the largest (0.59), followed by #20/40 (0.52), with #20/40(50%) exhibiting the lowest breakage degree, with only 0.39. After comparing the results, we confirm that #20/40(50%) can effectively reduce the proppant breakage degree by 5.8–33.9%.

**Fig. 14:**
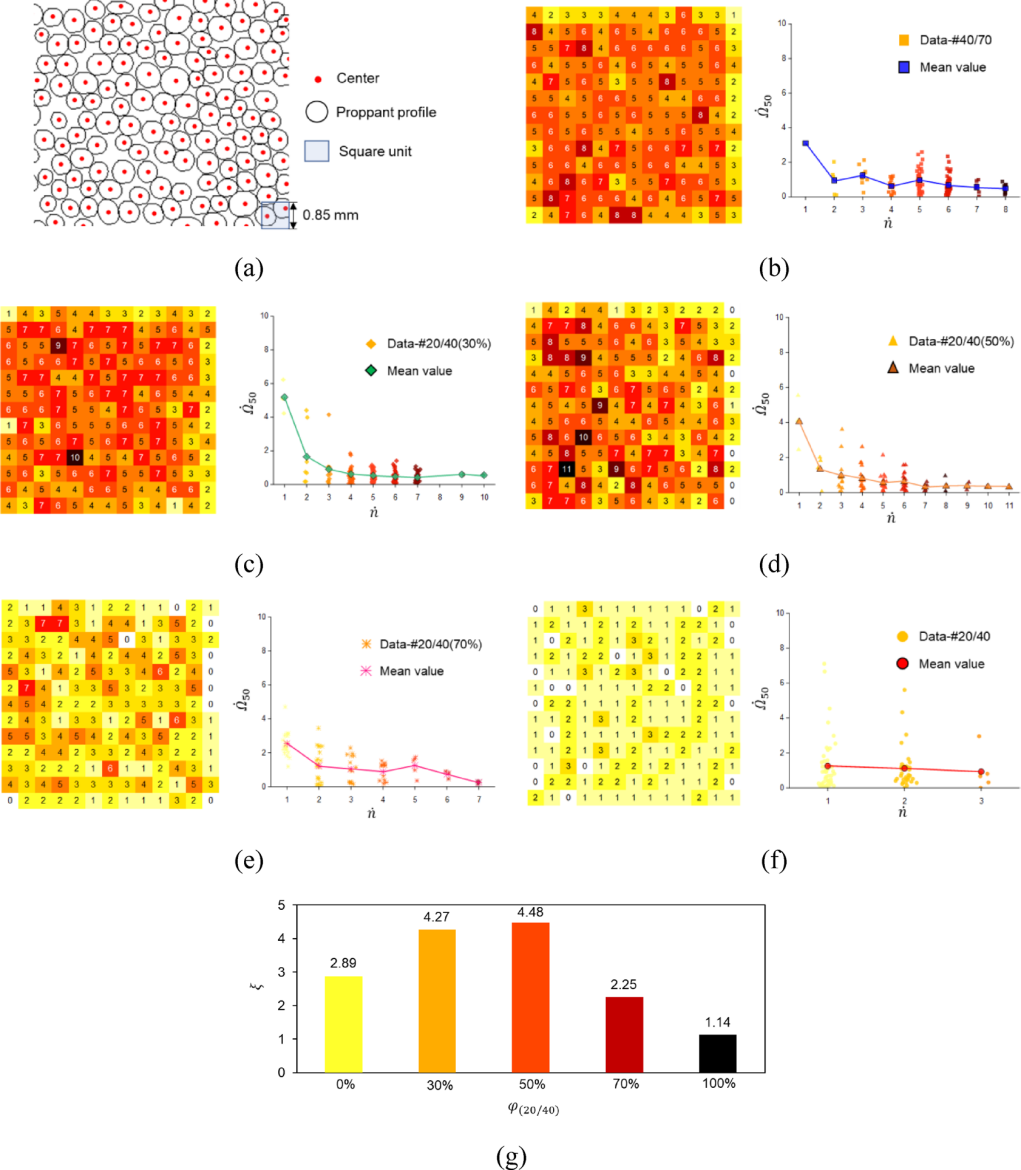
The relationship between $\dot{\Omega }_{50}$ and $\dot{n}$ obtained by statistical methods.

To reveal the non-uniform breakage behavior of the MP, we obtain and compare the breakage degrees of local regions at 50 MPa, denoted as $\dot{\Omega }_{50}$. We divide the entire microscope observation area into several local analysis units of the same size. As shown in Fig. 14a, we determine the number of proppant centroids $\dot{n}$ of square units at 0 MPa after executing image segmentation. We have established that the side length of a square unit is 0.85 mm, approximately equaling the diameter of 20 mesh proppant, which is done to ensure that the $\dot{n}$ of each unit is greater than or equal to 1. However, subsequent analyses will exclude some units with $\dot{n}$ = 0 at the edge of the image. Consequently, it is reasonable to infer that the proppant is more numerous and densely packed in the unit with a larger $\dot{n}$. In other words, it suggests that units with smaller $\dot{n}$ normally contain the large-size proppant.

Figure 14b–f show different relationships between $\dot{\Omega }_{50}$ and $\dot{n}$ of various patterns. Importantly, the mean value of $\dot{\Omega }_{50}$ for each $\dot{n}$ tends to decrease as $\dot{n}$ increases. In addition, when $\dot{n}$ increases between 1 and 3, the decrease in $\dot{\Omega }_{50}$ of the MP is greater than that of the SP. To compare quantitatively the non-uniform breakage of each specimen, we define the non-uniform proppant breakage coefficient $\xi$ which can be calculated as follows:

2 $$\begin{array}{*{20}c} {\xi = \frac{{{\text{max}}\left\{ {B\left( {\dot{n}} \right)} \right\}}}{{\frac{1}{{\dot{n}_{{{\text{max}}}} }}\mathop \sum \nolimits_{{\dot{n} = 1}}^{{\dot{n}_{{{\text{max}}}} }} B\left( {\dot{n}} \right)}} } \\ \end{array}$$

where $B\left( {\dot{n}} \right)$ is the mean value of $\dot{\Omega }_{50}$ corresponding to the given $\dot{n}$, $\dot{n}_{{{\text{max}}}}$ is the maximum of $\dot{n}$ obtained from the initial image segmentation. Generally, there is more significant non-uniform breakage if $\xi$ is larger in accordance with the definition.

Figure 14g demonstrates the value of $\xi$ for varying ratios of 20/40 mesh proppant ($\varphi_{{\left( {20/40} \right)}}$). The $\xi$ value for #20/40(50%) is the largest, indicating that the most significant non-uniform proppant breakage occurs at this pattern among five specimens. On the other hand, #20/40 with the smallest $\xi$ exhibit a comparatively more uniform breakage pattern.

**Fig. 15:**
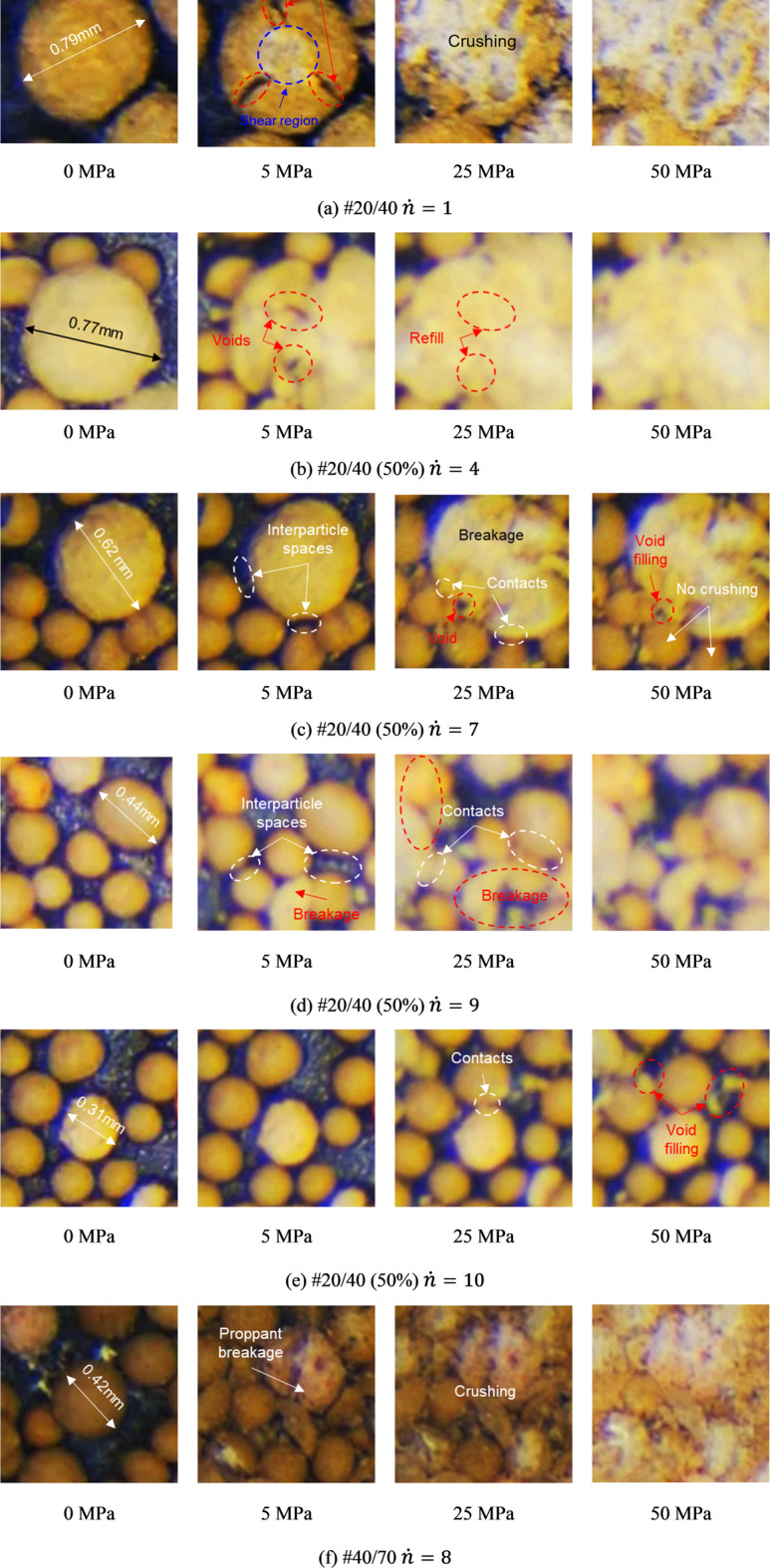
Proppant breakage images of local units.

We discuss the mechanism of proppant breakage by analyzing images with higher magnification. Without considering the embedment and matrix deformation, individual proppant axial compression is analogous to the scenario of contact between an elastic sphere and a rigid plate^39^. With the closure stress increasing, the contact between the proppant and sapphire induces a nearly circular contact area, which is shown in Fig. 15a at 5 MPa. According to the Hertzian contact mechanics, radial tensile stress is induced inside the contact area and exceeds its maximum value at the edge of the contact area^19^. Particularly, sand and uncoated ceramic proppant are brittle, and their failures typically follow the maximum tensile stress criterion. Thus, when the tensile stress at the edge reaches the material tensile strength, the tensile failure occurs, and fractures propagate along the proppant meridian. If the material is homogeneous and isotropic relatively, multiple tensile fractures occur, resulting in the “orange peel” failure. In addition, the point located approximately halfway down the radius of the contact area from its center has the largest shear stress, inducing a conical shear band is induced^40^. As a consequence, the mixed failure of tension and shear is the primary mode of proppant breakage.

After initial fracturing during the compression, the proppant still retains residual resistance. As the process of loading continues, more fractures are generated, and the failure mode undergoes a transition from localized breaking (such as exfoliating or splitting) to overall crushing (as shown in Fig. 15a at 25 MPa). The proppant breakage of #20/40 is more uniform, and almost all proppants are crushed (as shown in Fig. 15a at 50 MPa). Similarly, proppant crush is observed in #20/40 and 40/70 (Fig. 15a,f), with a significant amount of fine within interparticle pores.

For #20/40(50%), only a few large proppants have been broken within the unit. It can be observed from Fig. 15b that the closure stress continues to exert on the proppant fragments, inducing radial deformations following the breakage. Since proppants packed closely, the fragments of large proppants will contact the adjacent smaller proppants to fill the interparticle voids (Fig. 15c).

At 0 MPa, unbonded discrete proppants are only axially compressed, with no stress transferring by interparticle force chains. The non-negligible stress concentration effect in the small contact areas aggravates the failure. When the fragment generated by large proppant breakage deforms in the radial direction and contacts undamaged small proppants, a higher coordination number is generated, facilitating interparticle stress transfer. At the same time, the contact area becomes significantly larger while the stress concentration effect is eliminated. Small proppants, in conjunction with the fragments, construct a composite structure to resist closure stresses.

In addition, many local units contain solely 40/70 mesh proppants, as shown in Fig. 15d and e. When the closure stress reaches 25 MPa, a minority of proppants break down into fragments. However, at 50 MPa, the broken proppants are not crushed completely, which remains the resistance against closure stresses.

**Fig. 16:**
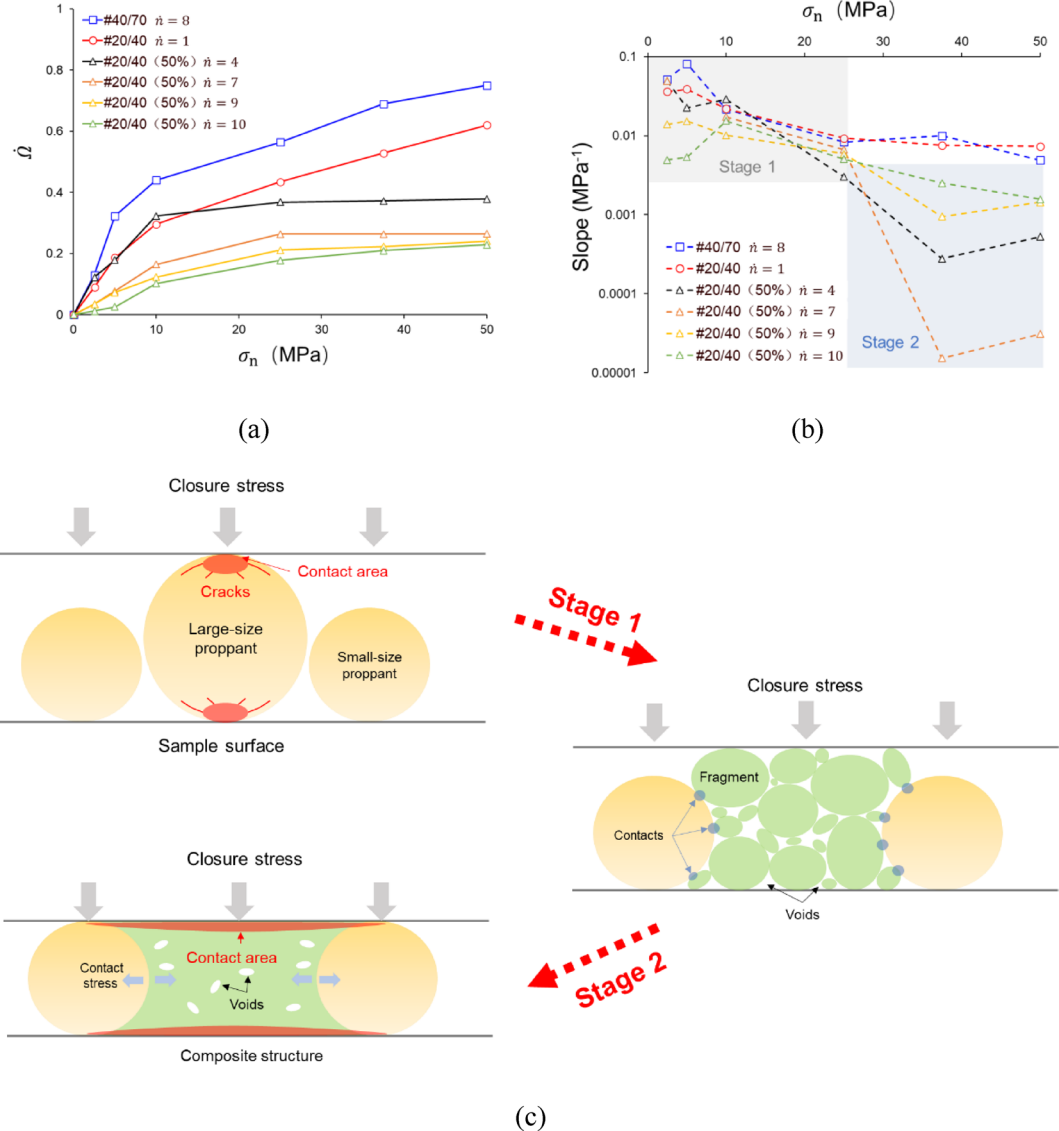
The two-stage process of partial composite structure in multi-size proppants.

To quantitatively contrast the evolution of local breakage behaviors, Fig. 16a illustrates the local breakage degree $\dot{\Omega }$ of the units in Fig. 15 corresponding to $\sigma_{{\text{n}}}$. Firstly, we observe a consistent variation trend among all units of #20/40(50%): the increment of $\dot{\Omega }$ obviously decreases as $\sigma_{{\text{n}}}$ increases. After 25 MPa, the increment of $\dot{\Omega }$ is close to 0. In contrast, although the increment of $\dot{\Omega }$ for the units of #20/40 and #40/70 is getting smaller with $\sigma_{{\text{n}}}$ loading, it is much larger than that of #20/40(50%). Figure 16b demonstrates that there is a significant difference in the slopes of $\sigma_{{\text{n}}}$ versus $\dot{\Omega }$ curves. It has been established that the slope values for all curves are similar before the application of 25 MPa, with a range of 0.005–0.08. After 25 MPa, a significant decrease in slope values in four curves with different $\dot{n}$ of #20/40 (50%) is observed, with a reduction of approximately 10–1000 times. The four $\dot{\Omega }$ versus $\sigma_{{\text{n}}}$ curves with different $\dot{n}$ of #20/40(50%) can be directly split into two stages based on their slopes, which are related to the behaviors of proppant breakage (Fig. 16c):

- Stage 1: For the units containing 20/40 mesh proppants, closure stress concentration occurs in the contact area of the large proppant, inducing its breakage into fragments, which $\dot{\Omega }$ increases significantly with the applied load. For the units without 20/40 mesh proppants, the increment of $\dot{\Omega }$ corresponding to $\sigma_{{\text{n}}}$ is relatively smaller.
- Stage 2: The fragmental 20/40 mesh proppants are crushed and deformed, contacting with adjacent proppants. This process ultimately forms the composite structure, creating large contact areas and generating interparticle stress transfers. Thus, the composite structure alleviates the stress concentration, under which small proppants in the structure are less likely to break. As a result, the increment of $\dot{\Omega }$ in response to $\sigma_{{\text{n}}}$ decreases, and the slope of this stage becomes much smaller. Moreover, partial composite structures akin to “strong pillars” play a pivotal role in resisting closure stresses and indirectly mitigating proppant breakage in other regions, which leads to a decrease in the slope of $\dot{\Omega }$ versus $\sigma_{{\text{n}}}$ curves for units without 20/40 mesh proppants as well.

In contrast, the slope of the #20/40 $\dot{n} = 8$ and #40/70 $\dot{n} = 1$ curves remain relatively larger, which are not distinguished by the presence of two stages. Consequently, the two-stage formation of the composite structure is necessarily coterminous with the non-uniform breakage of multi-size proppants. In order to form the composite structure, the non-uniform breakage is a prerequisite. In turn, the composite structure prevents the continued breakage of overall proppants, thus ensuring the maintenance of the non-uniform breakage.

### The deformation of proppant layer

**Fig. 17:**
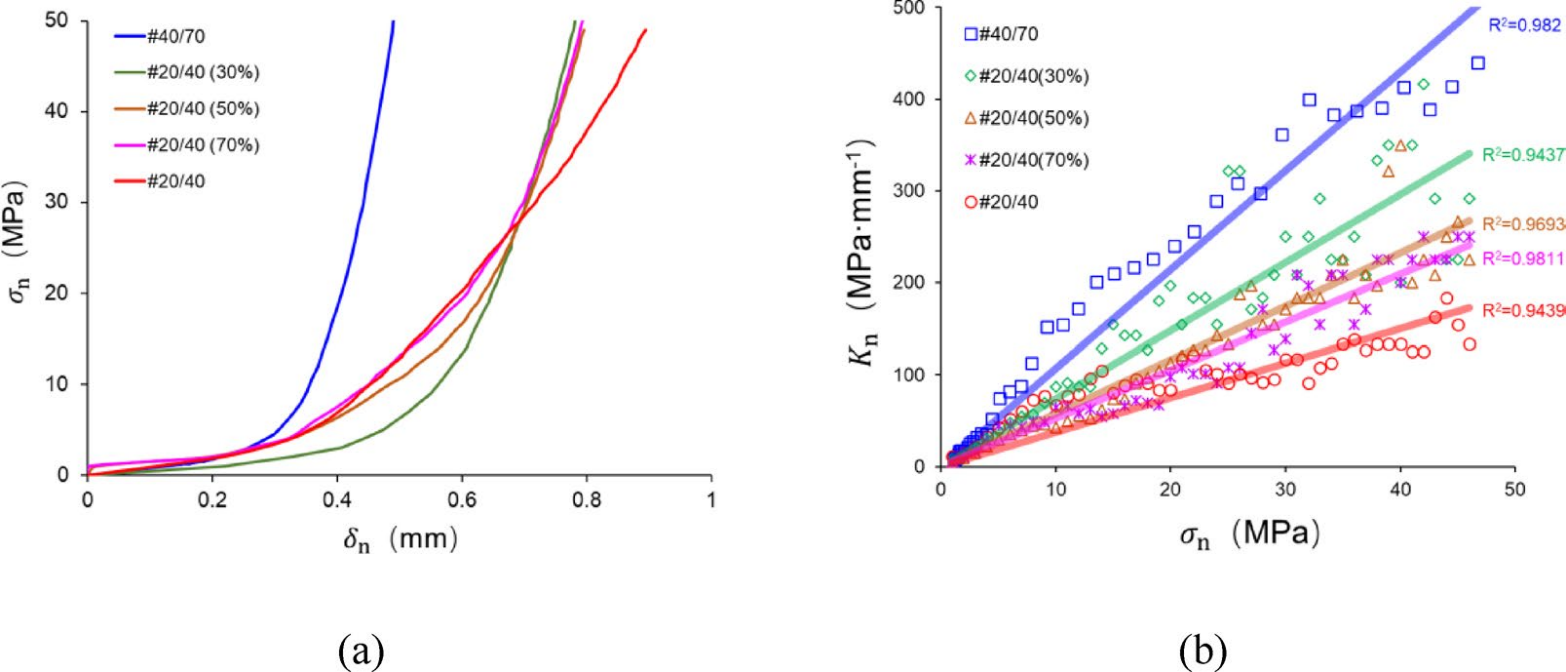
The deformation and normal stiffness of proppant layer induced by closure stresses.

Figure 17 shows the deformation of the proppant layer ($\delta_{{\text{n}}}$) corresponding to $\sigma_{{\text{n}}}$. with a highly nonlinear relationship between $\delta_{{\text{n}}}$ and $\sigma_{{\text{n}}}$. When $\sigma_{{\text{n}}}$ is low (< 5 MPa), the value of $\delta_{{\text{n}}}$ rapidly increases rapidly, and the slope of the curves is similar for several specimens. Furthermore, as $\sigma_{{\text{n}}}$ increases, the slope increases significantly. The nonlinear mechanical behavior is governed by proppant deformations and breakage^33^, resulting in different trends of the $\sigma_{{\text{n}}}$–$\delta_{{\text{n}}}$ relationship for various proppant placements. However, due to the presence of proppants, the aperture cannot be completely closed without embedment, and $\delta_{{\text{n}}}$ approximately reaches to the maximum proppant diameter under the high closure stress.

In order to capture the deformation gradient of the proppant layer, we introduce the concept of normal stiffness $K_{{\text{n}}}$. To illustrate, $K_{{\text{n}}}$ represents the closure resistance, which means the higher $K_{{\text{n}}}$, the smaller $\delta_{{\text{n}}}$ under the same closure stress. $K_{{\text{n}}}$ can be calculated as follows:

3 $$\begin{array}{*{20}c} {K_{{\text{n}}} = \frac{{d\sigma_{{\text{n}}} }}{{d\delta_{{\text{n}}} }}} \\ \end{array}$$

As illustrated in Fig. 17b, the variation in the relationship between $K_{{\text{n}}}$ and $\sigma_{{\text{n}}}$ is presented. The scatter points represent the stiffness values calculated from the experimental curves, while the straight line indicates the fitting result (Table 2). It is evident that as $\sigma_{{\text{n}}}$ increases, $K_{{\text{n}}}$ enhances significantly, essentially following the linear function (Eq. 4). Furthermore, there is a significant difference in the parameter of linear fitting ($\kappa$) of the five specimens, following the order: #40/70 > #20/40 (30%) > #20/40 (50%) > #20/40 (70%) > #20/40. The results indicate that the proppant layer presents a lower stiffness in correlation with an increase in the ratio of 20/40 mesh proppants in this study.

4 $$\begin{array}{*{20}c} {K_{{\text{n}}} = \kappa \sigma_{{\text{n}}} } \\ \end{array}$$

**Table 2:** The function of linear fitting for different specimens.

Sampe ID	Fitting function
#40/70	*y* = 10.743 *x*
#20/40(30%)	*y* = 7.403 *x*
#20/40(50%)	*y* = 5.807 *x*
#20/40(70%)	*y* = 5.244 *x*
#20/40	*y* = 3.749 *x*

**Fig. 18:**
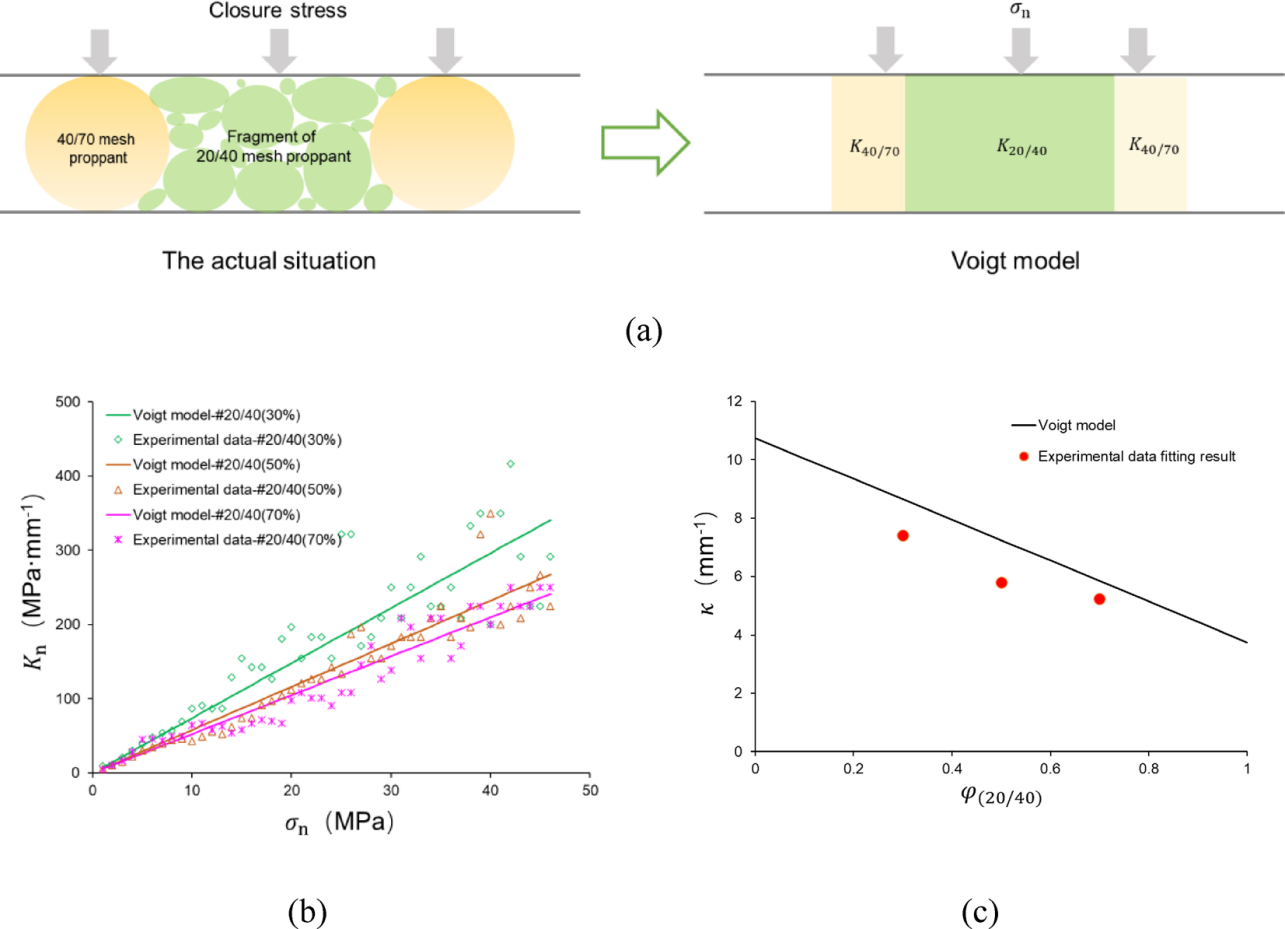
The comparison of Voigt model predictions and experimental data.

As the 20/40 mesh proppant breaks and the composite structure in the MP is formed, the fragment of the 20/40 mesh proppant and the 40/70 mesh proppant resist the closure stresses and deform in equal deformation. The proppant layer can be regarded as an equivalent medium, analogous to the scenario depicted by the Voigt model^41^ (Fig. 18a). The Voigt model is a classical theoretical framework for predicting the effective elastic modulus of multiphase composite materials, particularly suitable for characterizing the equivalent mechanical behavior of multicomponent rock systems^42,43^. This model operates under three fundamental assumptions: (a) All constituent materials experience identical strain in the same spatial direction; (b) The macroscopic stress of the composite material equals the volumetric-weighted average of stresses within individual components; (c) The material exhibits macroscale homogeneity when analyzed through a statistical continuum approach.

For the rock medium containing $n$ components, the equivalent elastic modulus $E_{{{\text{eq}}}}$ can be expressed as:

5 $$\begin{array}{*{20}c} {E_{{{\text{eq}}}} = \frac{{\sigma_{{\text{n}}} }}{\varepsilon } = \mathop \sum \limits_{i = 1}^{n} \phi_{i} E_{i} } \\ \end{array}$$

In Eq. (5), $\phi_{i}$ is the volume fraction of the *i*-th component, subject to the normalization condition $\sum\nolimits_{i = 1}^{n} {\phi_{i} } = 1$, and $E_{i}$ is its corresponding elastic modulus.

Analogously, the constitutive relationship for the closed stress-deformation behavior in multi-component media under uniform deformation conditions can be formulated. For a containing $n$ components, the equivalent stiffness $K_{{{\text{eq}}}}$ can be expressed as:

6 $$\begin{array}{*{20}c} {K_{{{\text{eq}}}} = \frac{{\sigma_{{\text{n}}} }}{{\delta_{{\text{n}}} }} = \mathop \sum \limits_{i = 1}^{n} \phi_{i} K_{i} } \\ \end{array}$$

where $K_{i}$ is the stiffness of the *i*-th component.

For the multi-size proppant placement investigated in the study, the equivalent stiffness of the multi-size proppant layer can be calculated under the specific closure stress using Eq. (7):

7 $$\begin{array}{*{20}c} {K_{{{\text{eq}}}} = \varphi_{20/40} K_{20/40} + \left( {1 - \varphi_{20/40} } \right) K_{40/70} } \\ \end{array}$$

where $K_{20/40}$ represents the normal stiffness of fragmental 20/40 mesh proppant, MPa mm^−1^; $K_{40/70}$ equals to the normal stiffness of 40/70 mesh proppant, MPa mm^−1^.

The variation rules of $K_{20/40}$ and $K_{40/70}$ corresponding to the closure stress are simplified to the linear fitting function of #20/40 and #40/70, and the correspondence between $K_{{{\text{eq}}}}$ and $\sigma_{{\text{n}}}$ of the MP can be calculated by Eq. (7) (Fig. 18b). It is worth noting that the application of Eq. (7) is an approximation requiring careful consideration of its underlying assumptions. Crucially, fragmental proppant particles transition into non-elastic continua exhibiting plastic behavior, thereby violating the fundamental elastic deformation premise of the Voigt model. Nevertheless, this simplified approach remains applicable to complex system analyses and retains practical prediction value in engineering contexts (Fig. 18b). The model demonstrates an ability to predict the overall trend of the variation of the proppant layer stiffness in the experiments, and the experimental results fluctuated around the values predicted values.

In addition, the gradient of the model curve depicted in Fig. 18b corresponds to the value of $\kappa$ as determined by the model. As illustrated in Fig. 18c, it is evident that all three specimens demonstrate a lower $\kappa$ in experiments than that predicted by the Voigt model. We speculate that the main factor that contributes to the error is: The simplified calculation of $K_{20/40}$ and $K_{40/70}$. The breakage characterization of the SP and MP is different. Particularly, a number of 40/70 mesh proppants remain unbroken in the MP, whereas almost all 40/70 mesh proppants in the SP are broken and crushed due to the closure stress. As a result, there is a discrepancy between the actual $K_{40/70}$ of the MP and the $K_{40/70}$ obtained from the simplified method.

### Characterizations of pores in the proppant layer

**Fig. 19:**
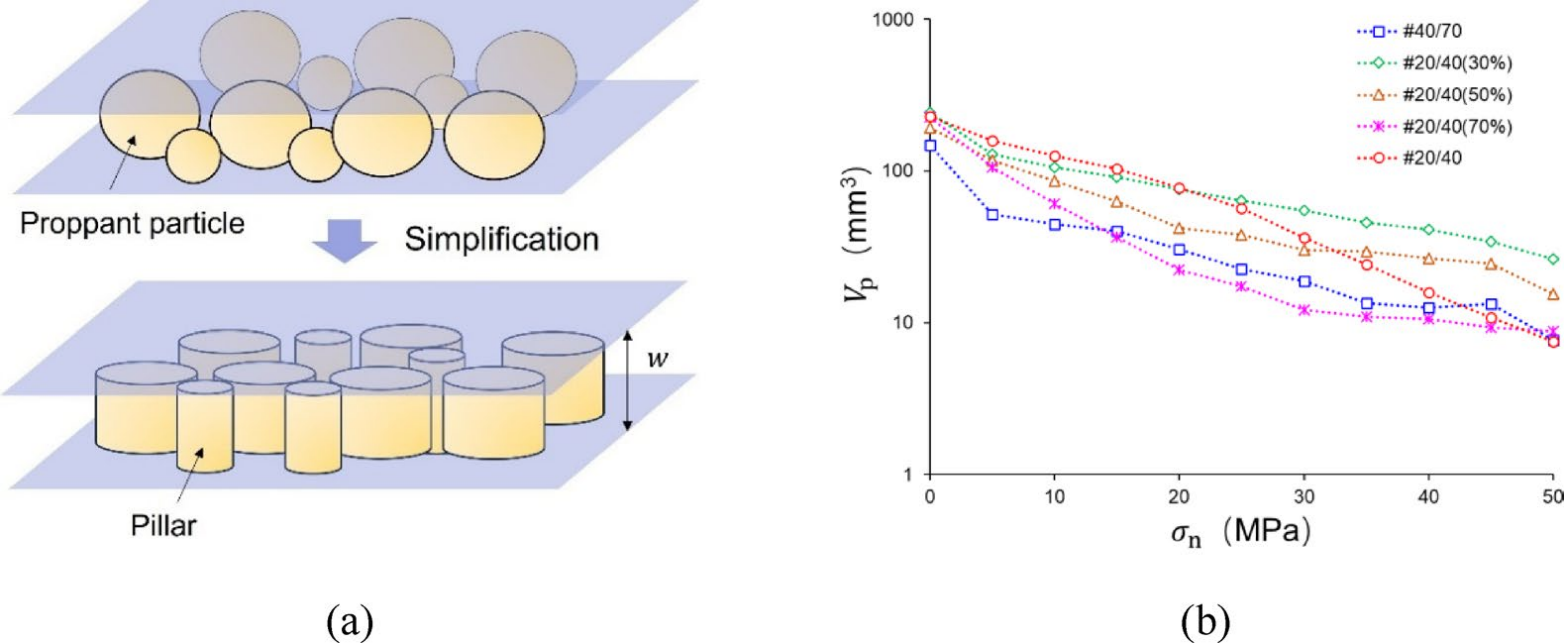
The simplification method to calculate pore volume $V_{{\text{p}}}$ and its variation corresponding to $\sigma_{{\text{n}}}$.

The pore volume and its connectivity directly influence the permeability of the proppant layer. As shown in images provided in “The process of proppant breakage” section, proppant deformation and breakage result in the damaged pore structure. Given the considerable difficulty of directly determining the pore volume of the proppant layer, this paper adopts a simplified method (Fig. 19a), whereby proppants are simplified into columns with the same height equal to the current propped aperture, which allows the 2D pore area to be obtained from the image calculations, acquiring the pore volume $V_{{\text{p}}}$ by multiplying by the current propped aperture to (Eq. 8).

8 $$\begin{array}{*{20}c} {V_{{\text{p}}} = \left( {A_{{\text{t}}} - A_{{\text{c}}} } \right) \times \left( {d_{{\text{m}}} - \delta_{{\text{n}}} } \right)} \\ \end{array}$$

where $V_{{\text{p}}}$ is the pore volume under the current closure stress, mm^3^; $A_{{\text{t}}}$ is the area of the observation by the microscope, mm^3^; $A_{{\text{c}}}$ is the proppant projection area at the current closure stress, which can be obtained through image analyses, mm^2^; $d_{{\text{m}}}$ represents the maximum diameter of proppants, being the initial aperture as well, which can be obtained from 3D scanning results (Fig. 5), mm; $\delta_{{\text{n}}}$ can be obtained by the LVDT, mm.

It is important to acknowledge the limitations of this simplified method which underestimates the pore volume, consequently leading to a relatively large error for the MP. However, when the elevated closure stress causes the proppant to be broken, the morphology of the fragmented proppant changes accordingly, from spherical to columnar. Therefore, the error of the simplified method decreases under conditions of high closure stresses.

Figure 19b demonstrates evolutions of the pore volume with the closure stress loading. At the initial condition (0 MPa), #20/40(30%) has the largest $V_{{\text{p}}}$ (243 mm^3^) while #40/70 has the smallest $V_{{\text{p}}}$ (147 mm^3^). Upon $\sigma_{{\text{n}}}$ reaching 50 MPa, #20/40(30%) exhibits the maximum $V_{{\text{p}}}$ (26.33 mm^3^), followed by #20/40(50%) with 15.41 mm^3^, #20/40(70%) with 8.81 mm^3^, #40/70 with 7.76 mm^3^, and #20/40 with 7.56 mm^3^. In summary, the pore volume of the multi-size proppant layer is greater than that of the single-size interval proppant layer under 50 MPa.

**Fig. 20:**
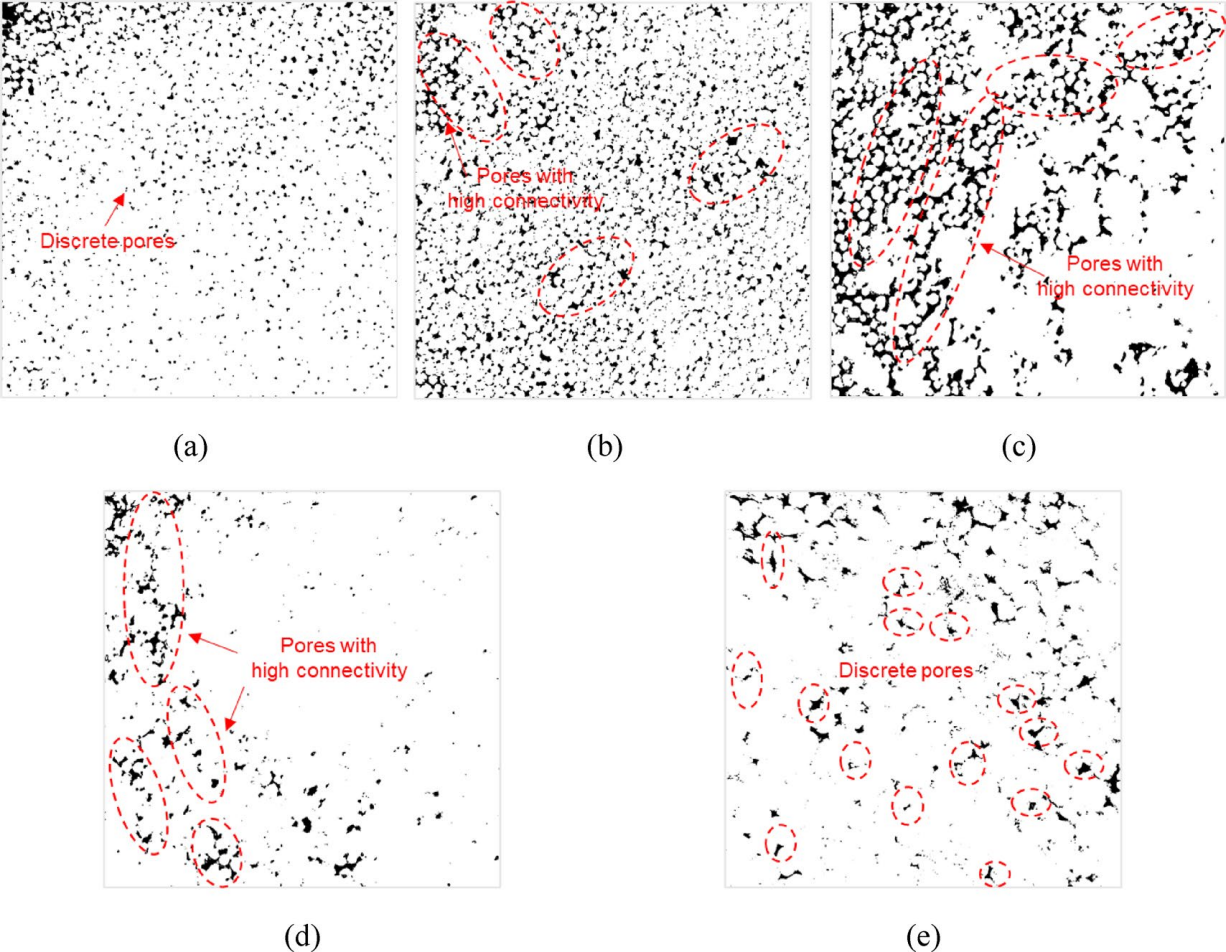
The binary images of each specimen at 50 MPa (White: proppants, black: pores): (**a**) #40/70 (**b**) #20/40(30%) (**c**) #20/40(50%) (**d**) #20/40(70%) (**e**) #20/40.

Furthermore, the permeability of porous media is not only dominated by the pore volume, moreover, the effect of pore structure, connectivity, and distribution should not be disregarded. Figure 20 shows the binary images of each specimen at 50 MPa. We can find that discrete pore distributions and low pore connectivity are induced by uniform proppant breakage in the SP (Fig. 20a,e). In contrast, large-size interparticle pores (Fig. 19b) with comparatively high connectivity are maintained in the MP, and the pore connectivity is optimized in #20/40(50%) among them.

**Fig. 21:**
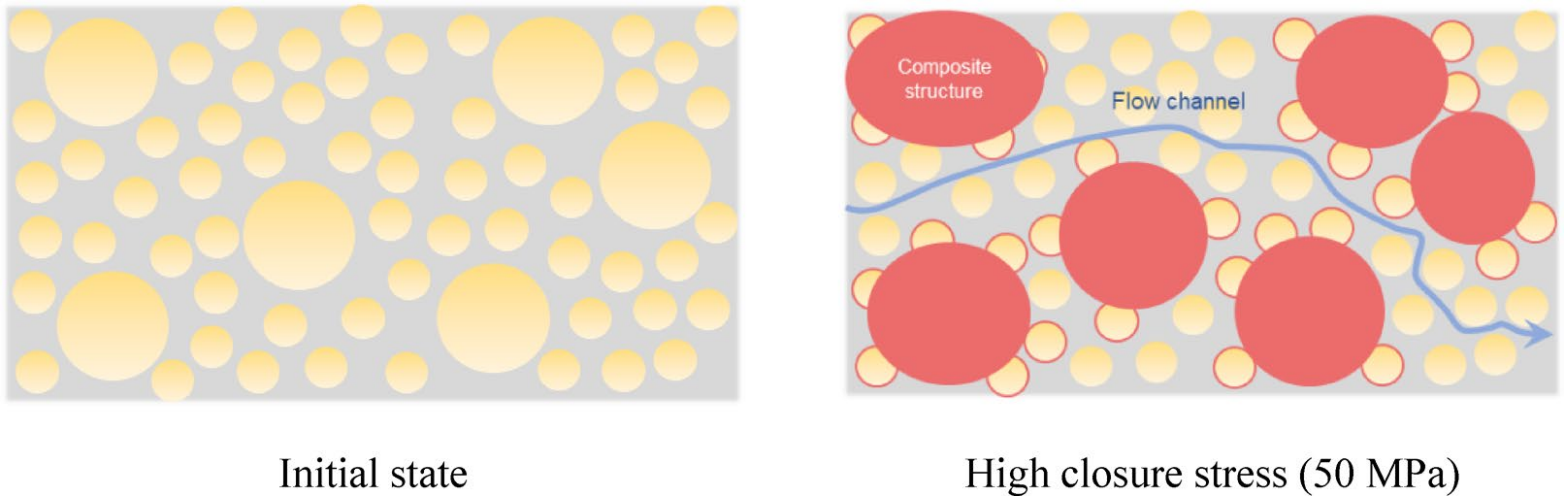
Schematic diagram of flow channels in the multi-size proppant layer.

In the porous media, the main flow channels consist of interconnected pores that exhibit high connectivity, in contrast to isolated pores that exert minimal influence on the effective permeability^44^. The reduced connectivity of these pores can be attributed to the relatively uniform proppant breakage and the higher breakage degree. In comparison, the multi-size proppants display more non-uniform breakage. Consequently, the formation of large interparticle pores with a high degree of connectivity, which in turn forms effective flow pathways (Fig. 21).

## Discussions

In this part, we discuss the limitations of the visual method and the validity of the above results. In additional experiments, 40/70 mesh proppant, 20/40 mesh proppant, and mixed proppants in the same volume ratio, were placed on the smooth granite discs, and designated #Granite-40/70, #Granite-20/40, and #Granite-20/40(50%), respectively. The procedures and condition settings utilized in the extra experiments were identical to those employed in previous experiments, and the mechanical parameters of the granite at 100 °C shown in Table 3. The granite specimens were taken from outcrops in Shandong, China.

**Table 3:** The mechanical properties (100℃) of the granite specimens.

Mechanical property	Value
Density (g/cm^3^)	2.63–2.89
Elastic modulus (GPa)	39–41
Poisson’s ratio	0.19–0.29
Uniaxial compress strength (MPa)	121–132

**Fig. 22:**
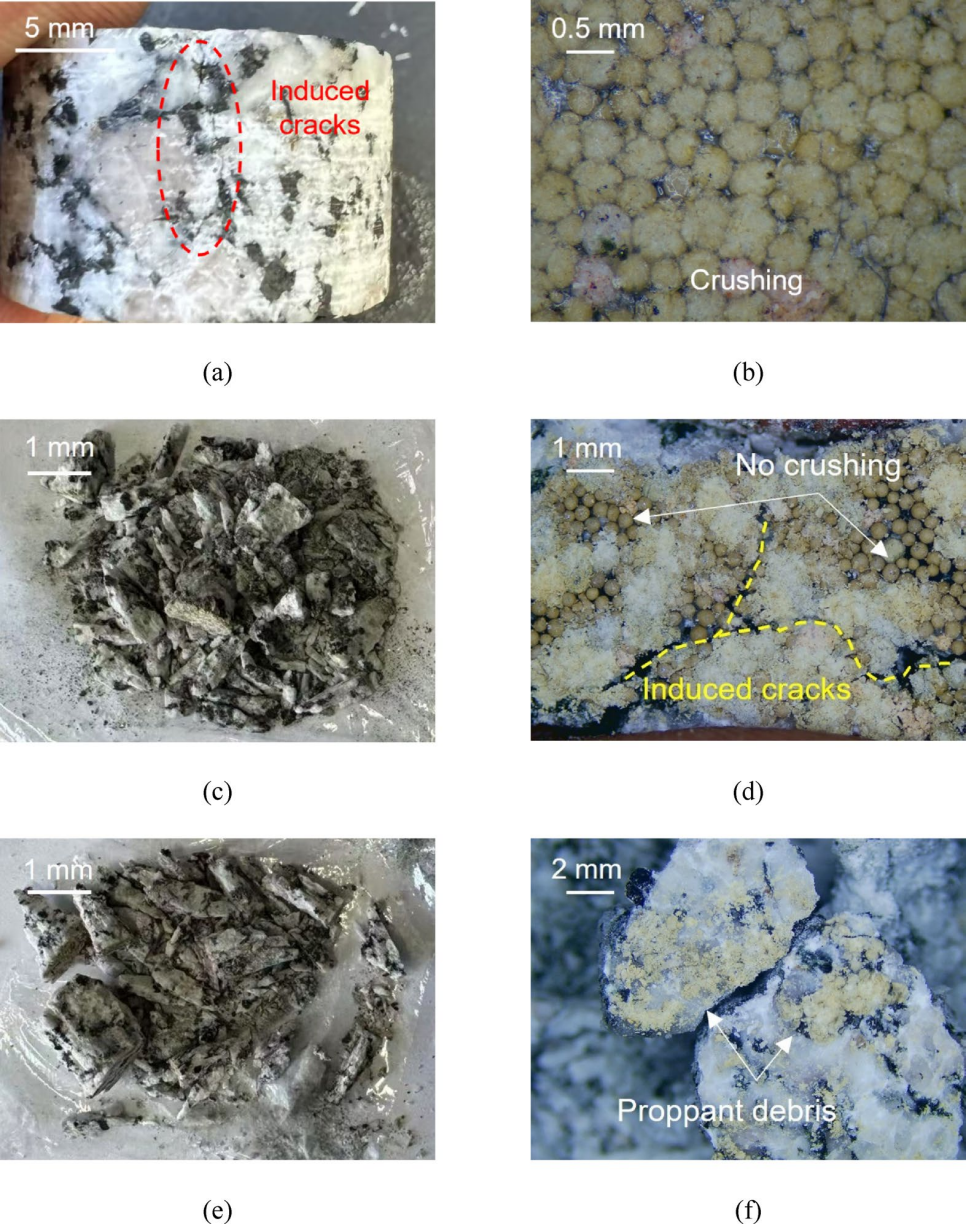
The photos of tested granite specimens and proppants. #Granite-40/70: (**a**) and (**b**); #Granite-20/40(50%): (**c**) and (**d**); #Granite-20/40: (**e**) and (**f**).

It was determined that #Granite-20/40 (50%) (Fig. 22c) and #Granite-20/40 (Fig. 22e) caused the brittle failure during the loading process, thereby rendering it impossible to exert closure stresses higher than 50 MPa. Conversely, only #Granite-40/70 was able to successfully complete the loading process, albeit with numerous induced fractures in the granite specimen (Fig. 22a). The application of higher closure stresses to brittle materials, such as granite, has been observed to result in the formation of stress concentrations within the proppant contact area, which, in turn, tends to cause brittle failure under no confining pressure conditions and experiment failure. Although the application of confining pressure has been demonstrated to enhance the strength of granite specimens^45,46^, it is challenging to achieve the necessary visualization requirements. Furthermore, the non-homogeneity of granite has a considerable effect on the experiment, which can lead to difficulties in replicating experimental results. Consequently, the utilization of specimens with proppant-steel contacts was deemed essential for the execution of the experiments.

The proppants on the surface of the granite specimens were observed after experiments. As illustrated in the Fig. 22, the proppant breakage occurs under the experimental conditions. Specifically, the 40/70 mesh proppant of the #Granite-40/70 exhibited the overall crushing (Fig. 22b), while the proppant on #Granite-20/40 demonstrating more severe breakage (Fig. 22f), resulting in the retention of only partial debris and no identifiable intact proppants were observed. Conversely, #Granite-20/40 (50%) exhibited the analogous phenomenon to #20/40(50%), where 20/40 mesh proppants are crushed, while the surrounding 40/70 mesh proppants remain relatively integrity (Fig. 22d). This result suggests that in multi-size proppants in contact with granite, the high closure stresses can induce the formation of composite structures. These structures assist in mitigating the breakage of 40/70 mesh proppant and potentially preserving highly permeable flow channels in areas of low breakage degree.

Furthermore, the effect of proppant embedment is examined in these experiments. By scanning the granite surface post-experiment and comparing the original morphology, it was determined that there were rarely embedment pits deeper than 0.1 mm. The two underlying reasons underpin the negligible proppant embedment. Firstly, the elastic modulus of the granite (39–41 GPa) is higher than that of the ceramic proppant (37.2 GPa). When the proppant contacts with the granite under closure stresses, the proppant exhibits a propensity to deform and fracture on its own rather than to embed itself in the granite. However, for rocks with high clay mineral contents, such as shales, mudstones, and sandstones, the proppants embed into the rocks firstly under high closure stresses. This indicates that the conclusions of this study are more applicable to deep hard-rock reservoirs than to soft-rock reservoirs. Secondly, the slight embedding of the proppant leads to the brittle failure of the specimens, indicating that the proppant is unable to continue embedding into the granite in the experiments.

**Fig. 23:**
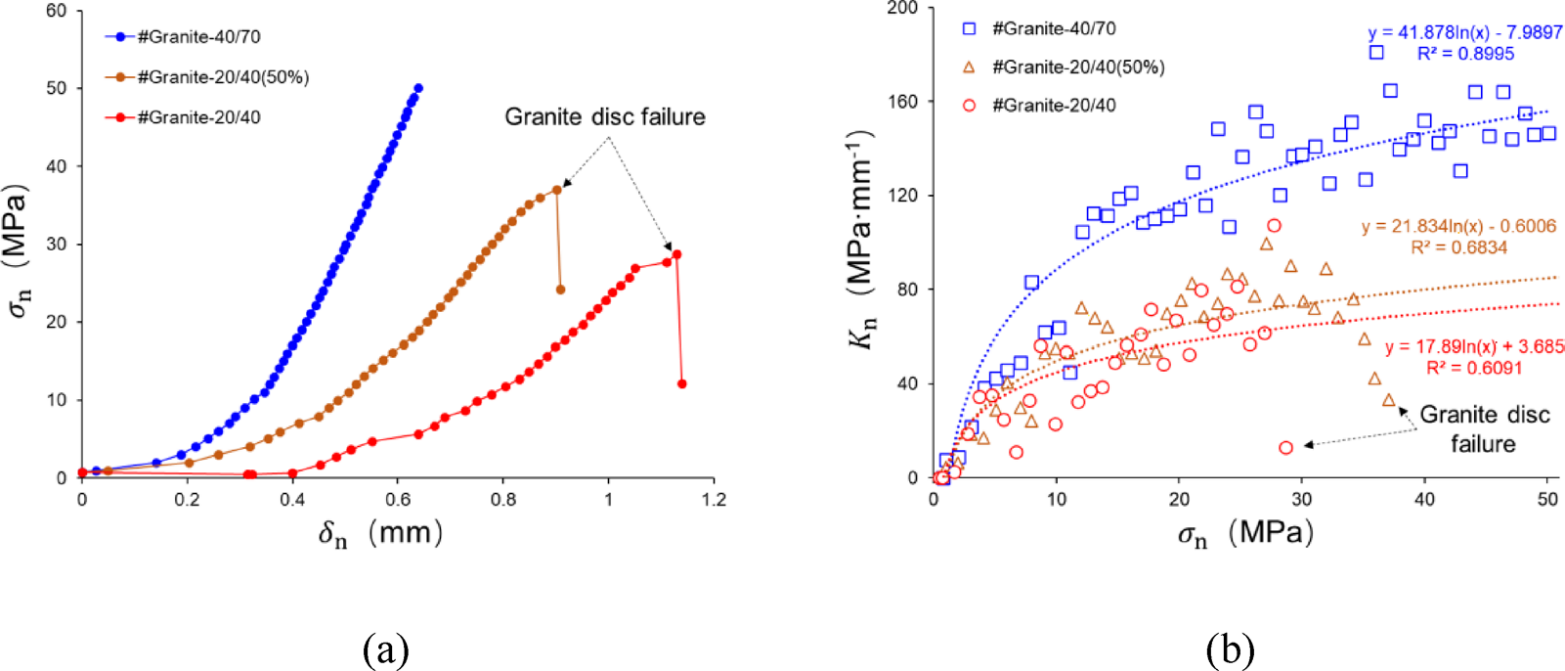
The variation of propped aperture and normal stiffness induced by closure stresses of granite specimens.

Finally, the closure behavior of propped fractures for granite-proppant specimens is examined. As demonstrated in Fig. 23a, the variation propped aperture of granite-proppant with closure stress is characterized as nonlinear. In contrast to the steel-proppant specimens, the $\delta_{{\text{n}}}$ of the granite-proppant specimens contains the deformation of the damaged granite specimen where microfractures in the granite are induced by stresses, resulting in a smaller stiffness than that of the steel-proppant specimens. Figure 23b demonstrates the nonlinear variation of the $K_{{\text{n}}}$ concerning the closure stress for the granite-proppant-placed specimen, in which the dashed line represents the fit result by logarithmic functions. However, it is noteworthy that there are two features that are consistent with the observed phenomenon in the steel-proppant specimens: (1) The $K_{{\text{n}}}$ of MP is positioned between that of the SP; (2) Prior to the specimen failures, the $K_{{\text{n}}}$ is increasing with the closure stress loading.

The findings of the additional experiments demonstrate that, in the case of hard rocks such as granite, there are significant breakages of both 20/40 mesh and 40/70 mesh ceramic proppants with low sand concentration under high closure stress conditions. Some results of the granite-proppant experiments are analogous to those of the steel-proppant experiments, wherein the breakage of multi-size proppant is non-uniform. However, in soft rock reservoirs, where the behavior of proppant-rock interaction is more complex, further research focusing on the proppant-rock interaction is anticipated to be investigated more comprehensively by enhancing the equipment and methods employed.

## Conclusions

Since there is a paucity of research in the process of multi-size proppant breakage under deep-hard reservoir conditions, we designed a visualization experiment to investigate the real-time processes of proppant breakage and proppant layer deformation under high temperature (100 °C) and high closure stress (0–50 MPa) conditions. The following conclusions offer insights into the multi-size proppant placement optimization in EGS:

1. The breakage degree is defined and utilized to quantitatively compare the severity of proppant breakage across different placed patterns. At the closure stress of 50 MPa, the overall breakage degree of single-size range proppant placements is higher than that of multi-size proppant placements.
2. Significant non-uniform breakage has been observed in the specimens with multi-size proppant placement. Hence the multi-size proppant layer can be divided into two regions: high breakage degrees and low breakage degrees. Particularly, large interparticle pores with high connectivity form dominant flow pathways in the regions of low breakage degree, potentially maintaining permeability under high closure stresses. In contrast, the proppant breakage in the single-size range proppant placed specimens is more uniform, which induces comparatively discrete pore distributions and low pore connectivity.
3. The two-stage failure process has been clarified from the local scale in the regions. In Stage 1, closure stress concentration occurs in the contact area of the large proppant, leading to its fragments. In Stage 2, the fragments are crushed and deformed, contacting with adjacent the small proppants. This process ultimately forms the composite structure, which alleviates the stress concentration and overall subsequent proppant breakage, thereby ensuring the maintenance of both non-uniform breakage and local high-connectivity porous structure.
4. Proppant breakage is regarded as a pivotal factor, contributing to the nonlinear variation of proppant layer deformation with closure stresses. The stiffness of the multi-size proppant-placed specimens is intermediate between that of the two single-size range proppant-placed specimens.
5. The applicability of the conclusions is evidenced by comparing granite-proppant experiments with identical conditions. The non-uniform breakage characterization of multi-size proppant in the granite-proppant experiments has been observed, which is a satisfying match with the steel-proppant experiments. However, in soft rock reservoirs, where the behavior of proppant-rock interaction is more complex, further research focusing on the interaction between proppant and rock is anticipated to be investigated more comprehensively by enhancing the equipment and methods employed.

## Data Availability

The datasets used and/or analyzed during the current study available from the corresponding author upon reasonable request.
